# Metabolic‐Redox Remodeling of Inflammatory Synovium by Mannose‐Modified Selenium Nanoparticles for Osteoarthritis Therapy

**DOI:** 10.1002/advs.78188

**Published:** 2026-10-06

**Authors:** Yu‐Qing Zou, Chen‐Hao Xu, Bo‐Wei Li, Leung Chan, Yi‐Di Xu, Han‐Bo Su, Rui Peng, Fang‐Ji Luo, Ya‐Chen Peng, Zhen‐Yan Li, Vincent Kam Wai Wong, Xiao‐Fei Zheng, Xing Ma, Zhen‐Gang Zha, Tianfeng Chen, Huan‐Tian Zhang

**Affiliations:** ^1^ Department of Bone and Joint Surgery The First Affiliated Hospital of Jinan University Key Laboratory of Regenerative Medicine of Ministry of Education Jinan University Guangzhou China; ^2^ Department of Chemistry Institute of Nanotechnology and Intelligence (inAI) College of Chemistry and Materials Science Jinan University Guangzhou China; ^3^ Dr. Neher's Biophysics Laboratory for Innovative Drug Discovery State Key Laboratory of Mechanism and Quality of Chinese Medicines Macau University of Science and Technology Macao China; ^4^ Sauvage Laboratory for Smart Materials School of Integrated Circuits Harbin Institute of Technology (Shenzhen) Shenzhen China

**Keywords:** glycolysis, metabolic reprogramming, osteoarthritis, redox homeostasis, selenium nanoparticles, synovial inflammation

## Abstract

Osteoarthritis (OA) progression is driven by persistent synovial inflammation, amplified by oxidative stress and enhanced glycolysis. Our previous study has demonstrated that glucose transporter 1 (GLUT1) plays a key role in initiating OA synovitis via regulation of glycolysis. Single‐cell RNA sequencing and tissue‐specific fluorescent reporter mice (*Prg4^GFPCreERt2^* and *Lyz2‐iCre;tdTomato*) reveal that GLUT1 marks a hyperinflammatory synovial state coupled to impaired selenoprotein‐dependent redox homeostasis. Accordingly, mannose‐modified selenium nanoparticles (M‐SeNPs) are developed as an intra‐articular nanotherapeutic targeting hypermetabolic synovial niches while restoring redox balance and reprogramming dysregulated metabolism. M‐SeNPs are successfully synthesized and characterized, demonstrating preferential uptake by the GLUT1^high^ cells. Proteomic analysis indicates that the robust anti‐inflammatory activity of M‐SeNPs stems from coordinated metabolic reprogramming and reconstitution of the endogenous antioxidant system, driven by alterations in glycolytic protein and multiple selenoproteins. In vivo, studies demonstrate that, M‐SeNPs selectively accumulate in inflamed synovial tissues, effectively alleviating OA‐associated pain and restoring joint function. Histological and immunofluorescence analyses further demonstrate pronounced chondroprotection efficacy of M‐SeNPs in OA mouse models, which is redominantly mediated by metabolic rewiring and redox homeostasis restoration in the GLUT1^high^ synoviocytes. Collectively, our results establish M‐SeNPs as a compelling candidate for OA treatment via selective modulation of synovial metabolic pathway.

## Graphical abstract

Elevated GLUT1‐driven glycolysis and disrupted selenoprotein‐dependent redox homeostasis drive osteoarthritic progression. Intra‐articular mannose‐modified selenium nanoparticles (M‐SeNPs) selectively target hypermetabolic synovial cells to reprogram aberrant glycolysis and restore endogenous antioxidant defenses. This nanotherapeutics suppresses synovial inflammation, alleviates OA pain, and confers robust chondroprotection, effectively attenuating osteoarthritic pathology.

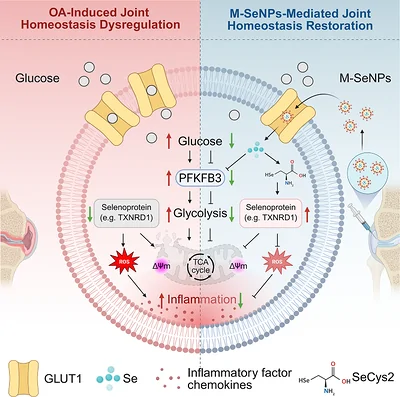

## 1 Introduction

Osteoarthritis (OA) has historically been considered as a disease of articular cartilage degeneration but is now increasingly recognized as a chronic inflammatory disorder [1]. Within the synovium, fibroblast‐like synoviocytes (FLS) and macrophages, are identified as the principal cell types driving the dysregulation of joint barriers and perpetuating inflammation [2]. For instance, activated FLS are pivotal to OA progression, as they secrete a suite of pro‐inflammatory cytokines, chemokines, and proteolytic enzymes that degrade joint integrity [3, 4, 5]. Concurrently, macrophage polarization toward the pro‐inflammatory M1 phenotype further exacerbates inflammation and impairs tissue repair [6, 7, 8]. These processes form a self‐reinforcing cycle: synovial inflammation promotes M1 macrophage polarization, and M1‐derived mediators can also activate FLS, forming a vicious cycle that drives chronic inflammation [9, 10, 11]. Therefore, a therapeutic strategy that simultaneously targets synovial inflammation and macrophage polarization represents a promising disease‐modifying approach.

In recent years, metabolic reprogramming, particularly the abnormal activation of glycolysis, has been identified as a critical driver perpetuating synovial inflammation. OA FLS exhibit a metabolic shift from mitochondrial oxidative phosphorylation toward glycolysis [12]. This enhanced glycolytic activity in FLS further drives synovial macrophage infiltration and polarization, thereby amplifying the inflammatory response. The initiation and sustained activity of glycolysis largely depend on effective glucose uptake [10]. The highly conserved major glucose transporter GLUT1 [13] plays a crucial facilitative role by maintaining a steady supply of glucose in various cancers [14, 15, 16] and inflammatory diseases [17, 18, 19, 20]. In addition, glycolytic flux is also tightly controlled by several rate‐limiting steps, for instance, critically controlled by 6‐phosphofructo‐2‐kinase/fructose‐2,6‐bisphosphatase (PFKFB) via fructose‐2,6‐bisphosphate (F‐2,6‐BP) synthesis [21]. Inhibition of PFKFB3 reduces glucose uptake and lactate secretion in rheumatoid arthritis FLS and ameliorates synovitis and joint destruction in a murine arthritis model [22]. Targeting aberrant glycolysis of FLS is promising for inhibiting OA inflammatory cascade.

Recent studies have linked the increased glycolytic flux to excessive ROS production through metabolite accumulation and mitochondrial suppression under inflammatory conditions. This ROS surge, which inflicts direct oxidative damage and acts as a pro‐inflammatory signal, establishes a maladaptive glycolysis‐ROS‐inflammation loop that sustains synovitis [23, 24, 25]. Selenoproteins, as vital components of the intracellular antioxidant system, exert cytoprotective effects by neutralizing and scavenging ROS through the catalytic activity of their active center's selenocysteine (Sec) residue [26, 27, 28]. Our previous work has established that selenium (Se) supplementation exerts protective effects in various inflammatory diseases by upregulating selenoprotein expression and activity [29, 30, 31, 32]. Kashin‐Beck disease serves as a primary clinical archetype of selenium‐deficiency‐driven osteoarthropathy, corroborating the causal relationship between selenium depletion and joint degeneration [33]. Accordingly, Se supplementation contributes to cartilage protection during OA progression [31, 34]. However, its effects on synovial inflammation and the underlying mechanisms remain largely unclear.

In this study, we have successfully developed mannose‐modified selenium nanoparticles (M‐SeNPs) as an intra‐articular nanotherapeutic targeting OA synovial niches. M‐SeNPs preferentially accumulate in inflamed synovium, exhibit potent anti‐inflammatory effect both in vitro and in vivo, therefore significantly ameliorate OA progression by metabolic reprogramming and redox homeostasis in the GLUT1^high^ synoviocytes.

## 2 Results

### 2.1 Enhanced Glycolysis Correlates to Impaired Selenoprotein System in OA

Our previous single‐cell RNA sequencing (scRNA‐seq) analysis demonstrated that enhanced glycolysis in the FLS promotes synovial macrophage infiltration and polarization in diabetic osteoarthritis (DOA) [10]. To further characterize the metabolic landscape of OA synovium, we performed scRNA‐seq analysis of additional OA samples. (Figure S1A). Clustering analysis identified seven major cell populations, including fibroblast‐like synoviocytes (Syn), macrophages (Mac), dendritic cells (DC), endothelial cells (EC), lymphocytes (LC), mast cells (Mast), and pericytes (PC) (Figure 1A–C and Figure S1B). Among these populations, FLS and macrophages exhibited prominently elevated glycolytic activity in OA synovium, accompanied by increased expression of glucose transporter and glycolytic genes, including *SLC2A1* (GLUT1) and *PFKFB3* (Figure 1C and Figure S1C–E).

**FIGURE 1:**
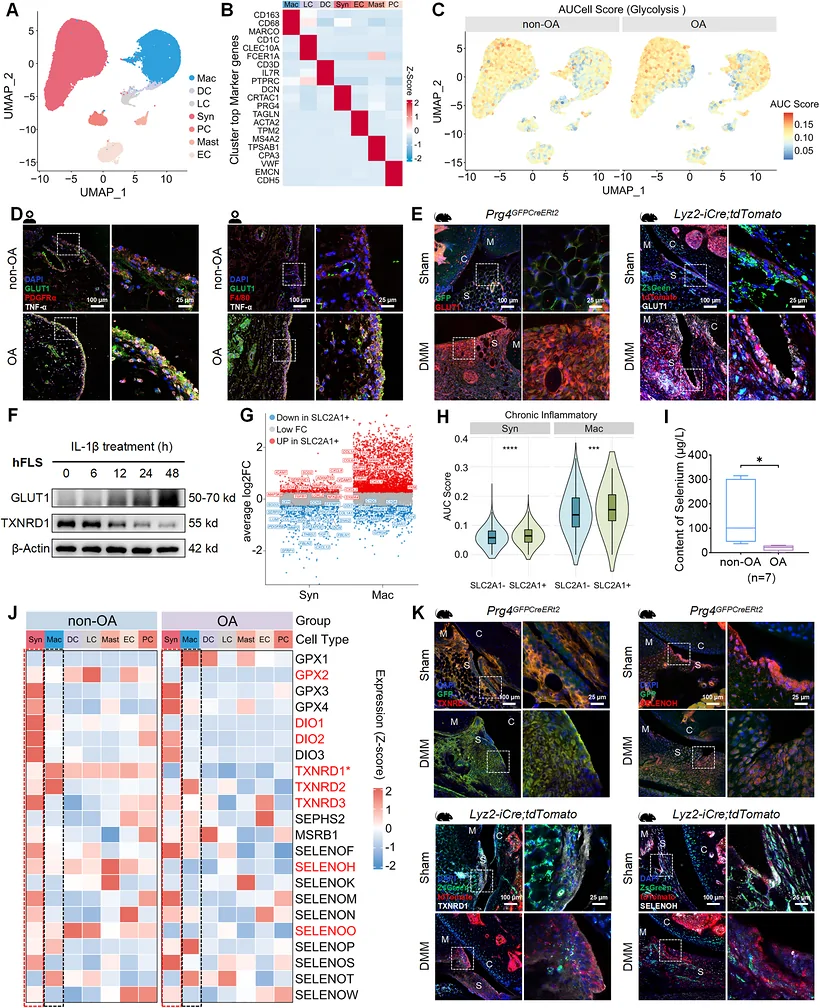
Inverse correlation between GLUT1 expression and selenoprotein homeostasis in OA synovium. (A) The Uniform Manifold Approximation and Projection (UMAP)‐based clustering identified seven distinct cell populations in control and OA synovium. The clusters were color‐coded for differentiation, with identities labeled on the right side of the plot. (B) Heatmap visualization of marker genes distinguishing the different cell populations. (C) Feature plots illustrating the glycolysis pathway scores calculated via AUCell. (D) Representative immunofluorescence images of human knee synovium from non‐OA and OA donors showing GLUT1 expression in distinct synovial cell populations. Left panels show co‐staining of GLUT1 with PDGFRα and TNF‐α to identify inflamed FLS, whereas right panels show co‐staining of GLUT1 with F4/80 and TNF‐α to identify pro‐inflammatory macrophages. Nuclei were counterstained with DAPI. (E) Representative immunofluorescence images showing GLUT1 expression in lineage‐traced GFP^+^ FLS and tdTomato^+^ macrophages. (F) Western blotting analysis showing time‐dependent changes in GLUT1 and TXNRD1 expression in FLS following IL‐1β stimulation. β‐Actin serves as a loading control. (G) Differential gene expression analysis between SLC2A1^+^ and SLC2A1^−^ subsets visualized via a volcano plot. (H) Violin plots showing the chronic inflammatory pathway scores, calculated via AUCell, in SLC2A1^+^ and SLC2A1^−^ cell subpopulations. (I) Selenium levels in synovial fluid from non‐OA and OA patients. (J) Heatmap of relative expression (z‐score) of selenoprotein‐related genes across synovial cell subpopulations derived from scRNA‐seq data. (K) Representative immunofluorescence images and showing TXNRD1 and SELENOH expression in GFP^+^ FLS and tdTomato^+^ macrophages. Syn: fibroblast‐like synoviocytes, Mac: macrophages, DC: dendritic cells, EC: endothelial cells, LC: lymphocytes, Mast: mast cells, PC: pericytes. Quantitative data in (I) are shown as mean ± S.D., with statistical significance determined by student's t‐test. *p < 0.05.

Immunofluorescence staining of human OA synovium and lineage‐traced DMM‐induced mouse models, together with in vitro experiments, consistently confirmed increased GLUT1 expression in activated FLS and macrophages (Figure 1D‐F and Figures S2–S4). Further pseudotime analysis suggested that increased *SLC2A1* expression was associated with pathological transition of synovial cell states during OA progression (Figure S5A–E). Subsequent analysis revealed that SLC2A1^+^ synovial cells displayed enhanced inflammatory, glycolytic, and fibrotic signatures compared with SLC2A1^−^ populations (Figure 1G, H and Figure S5F–J), suggesting that GLUT1^high^ cells represent a metabolically activated inflammatory state in OA.

Excessive glycolysis is closely linked with oxidative stress. The protective role of selenoproteins, key regulators of redox homeostasis, have been well documented in OA cartilage [35, 36], but remain incompletely elucidated in synovium. Consequently, we next investigated alterations in the synovial selenoprotein‐associated redox system. Oxidative stress scores were elevated in OA synovium, particularly in FLS and macrophages (Figure S6A, B). Mass spectrometry revealed reduced selenium levels in OA synovial fluid (Figure 1I), while scRNA‐seq analysis demonstrated broad dysregulation of selenoprotein‐related genes, including antioxidant regulators *TXNRD1* and *SELENOH* [37, 38, 39, 40, 41, 42] across multiple synovial populations (Figure 1J and Figure S6C). Consistently, TXNRD1 and SELENOH expression was decreased in IL‐1β‐stimulated FLS and DMM‐induced mouse synovium (Figure 1F, K and Figure S7). Interestingly, although selenium metabolism pathways were activated in OA synovium, SLC2A1^+^ cells exhibited reduced TXNRD1/SELENOH expression accompanied by increased oxidative stress (Figure S6D‐G). Spatial analysis further demonstrated an inverse association between GLUT1 and TXNRD1/SELENOH expression (Figure S8).

### 2.2 Design, Characterization, and Physicochemical Properties of M‐SeNPs

Given the elevated expression of GLUT1 in the inflamed synoviocytes including FLS and M1‐polarized macrophages. We designed Mannose‐modified‐SeNPs (M‐SeNPs) to specifically target these inflamed cells which aim to facilitate GLUT1^high^ cellular uptake (Figure 2A) [43]. Spherical SeNPs and M‐SeNPs with uniform size were synthesized at room temperature using a liquid‐phase stirring method. Energy‐dispersive spectroscopy (EDS) and transmission electron microscopy (TEM) demonstrated uniform spherical morphology of both selenium‐nanoparticles, while mannose modification improved nanoparticle dispersibility and reduced aggregation (Figure 2B, C). Dynamic light scattering (DLS) analysis revealed that M‐SeNPs exhibited an average hydrodynamic diameter of approximately 100 nm, comparable to that of SeNPs (Figure 2D, left). The successful grafting of mannose was evidenced by a shift in zeta potential from −2.7 to −1.2 mV (Figure 2D, right). This discrepancy likely resulted from the grafted mannose corona and its tightly bound hydration layer, which increase the effective hydrodynamic volume in aqueous media, thus compensating for the reduced core size under vacuum dehydration [44, 45].

**FIGURE 2:**
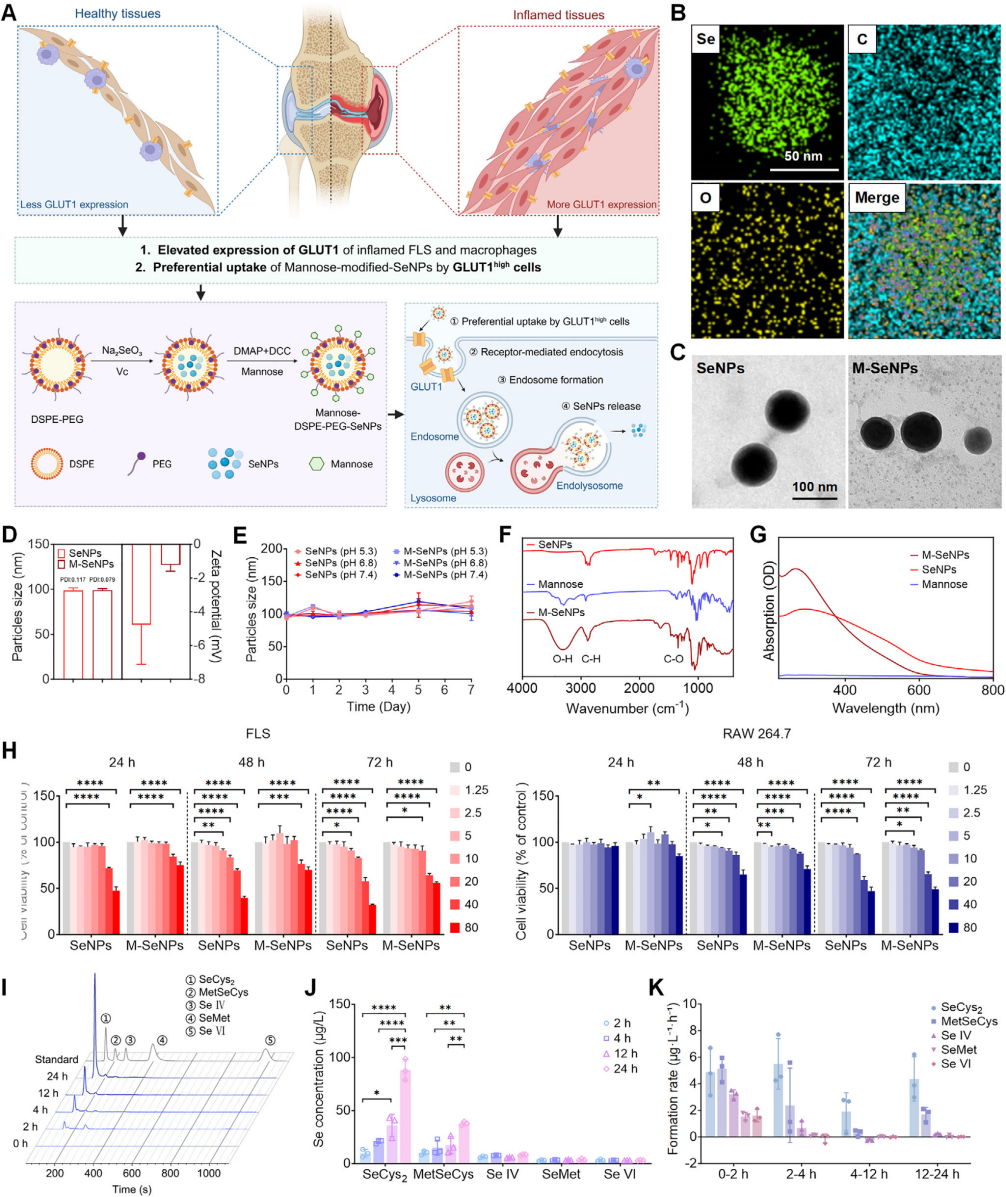
Design, synthesis, and physicochemical characterization of M‐SeNPs. (A) Schematic illustration of the therapeutic concept. GLUT1 over‐expression on inflammatory FLS and macrophages in osteoarthritis enables efficient delivery and uptake of mannose‐modified selenium nanoparticles (M‐SeNPs). (Created in BioRender.) (B) Representative TEM images of unmodified SeNPs and M‐SeNPs. (C) EDS confirming Se as the dominant elemental component of both nanoparticles. (D) DLS analysis showing the hydrodynamic diameters of SeNPs and M‐SeNPs. Zeta potential shifts from ‐2.7 mV to ‐1.2 mV following mannose functionalization, indicating successful surface modification. (E) Colloidal stability of SeNPs and M‐SeNPs evaluated by DLS over 7 days under physiologically relevant pH conditions (pH 5.3, 6.8, and 7.4). (F) FTIR spectra of M‐SeNPs showing characteristic O‐H stretching (∼3300 cm^−^^1^), C─H stretching (2800–3000 cm^−^^1^) and C‐O stretching (∼1000‐1100 cm^−^^1^), consistent with mannose conjugation. (G) UV–Vis absorption spectra of SeNPs and M‐SeNPs. (H) Cell viability of FLSs and RAW 264.7 macrophages following treatment with SeNPs and M‐SeNPs for 24, 48 and 72 h, as measured by CCK‐8 assay. Values were normalized to the untreated control (100%). (I) HPLC‐ICP‐MS analysis demonstrating intracellular metabolic conversion of M‐SeNPs into bioactive selenium species, predominantly SeCys_2_, within 24 h. (J) Concentrations of five selenium species (SeCys_2_, MetSeCys, Se IV, SeMet, and Se VI) determined at 2, 4, 12, and 24 h after incubation. (K) Calculated formation rates of individual selenium species across four incubation intervals (0–2 h, 2–4 h, 4–12 h, and 12–24 h), illustrating the dynamic transformation kinetics of intracellular selenium. DMAP: 4‐dimethylaminopyridine; DCC, N,N’‐Dicyclohexylcarbodiimide; Vc: Vitamin C, Ascorbic acid; DSPE: 1,2‐Distearoyl‐sn‐glycero‐3‐phosphorylethanolamine; PEG: Polyethylene glycol; C, Carbon; O, Oxygen. Quantitative data in (D, E, H, J, K) are shown as mean ± S.D., with statistical significance determined by one‐way ANOVA. *p < 0.05, **p < 0.01, ***p < 0.001, ****p < 0.0001.

The stability of SeNPs and M‐SeNPs was tracked for 7 days at pH 5.3, 6.8, and 7.4 to mimic lysosomal, inflammatory, and physiological environments. Both nanoparticles exhibited excellent stability, maintaining a consistent size of approximately 100 nm across all tested environments (Figure 2E). The successful grafting of mannose was evidenced by a shift in zeta potential from −2.7 to −1.2 mV (Figure 2D, right). Fourier‐transform infrared (FTIR) spectroscopy further revealed signature O‐H (∼3300 cm^−^^1^), C‐H (2800–3000 cm^−^^1^) and C‐O (1000–1100 cm^−^^1^) stretching vibrations characteristic of the carbohydrate moiety. The absence of a distinct C═O peak observed (1700–1740 cm^−^^1^) is consistent with the predominance of the cyclic hemiacetal structure of mannose over its transient open‐chain aldehydic isomer (Figure 2F) [46, 47, 48]. UV–Vis spectroscopy showed similar profiles (Figure 2G), though M‐SeNPs exhibited higher absorption peaks in the 200–400 nm range. This intensification attributed to mannose‐induced stabilization, which prevents aggregation and maintains high dispersion [49, 50]. Collectively, these findings underscore the rational design and successful synthesis of M‐SeNPs, characterized by their precise structural integrity and well‐defined physicochemical features.

The cytotoxicity of SeNPs and M‐SeNPs was evaluated via Cell Counting Kit‐8 (CCK‐8) assays as indicated. Both nanoparticles exhibited concentration‐ and time‐dependent effects on cell viability in FLS and RAW 264.7 macrophages. At 24 h, concentrations ≤ 20 µM showed minimal cytotoxicity, with cell viability remaining above 95%, whereas significant cytotoxicity was observed at a higher concentration, this cytotoxic trend was further exacerbated at 48 and 72 h (Figure 2H). Accordingly, 20 µM was selected as the maximum working concentration for subsequent experiments, remaining well above the cytotoxicity threshold (70%) according to ISO 10993–5:2009.

Since selenium has been reported to execute its functions through the synthesis of a series of selenoproteins in the cells [51], we then analyzed the intracellular fate and biotransformation of selenium following SeNPs treatment in FLS by High Performance Liquid Chromatography ‐Inductively Coupled Plasma Mass Spectrometry (HPLC‐ICP‐MS). Following M‐SeNPs treatment, intracellular selenium was rapidly converted into bioactive metabolites, including selenocystine (SeCys_2_) and methylselenocysteine (MetSeCys), whereas inorganic selenium intermediates were transiently detected and subsequently decreased (Figure 2I–K). These results indicate efficient intracellular selenium processing and support the potential of M‐SeNPs as a selenium delivery platform for restoring selenoprotein‐associated biological functions.

### 2.3 M‐SeNPs Exert Anti‐Inflammatory and Antioxidant Functions in GLUT1^high^ Cells

To investigate whether mannose modification enhances the cellular uptake of SeNPs by the inflamed cells, nanoparticles were labeled with the fluorescent dye 1,1'‐Dioctadecyl‐3,3,3',3'‐Tetramethylindodicarbocyanine,4‐Chlorobenzenesulfonate Salt (DiD). Both SeNPs and M‐SeNPs were effectively internalized by FLS and RAW 264.7 macrophages through a dose‐ and time‐dependently manner. M‐SeNPs exhibited significantly higher intracellular accumulation in both cell types relative to unmodified SeNPs. (Figure 3A, B, and Figure S9A, B). After pretreatment with GLUT1 inhibitor BAY‐876 (Figure S9C–E) [52, 53]or competitive incubation with free mannose [43], the uptake of M‐SeNPs by FLS and RAW264.7 cells was significantly reduced (Figure S9F, G), suggesting that GLUT1 and mannose recognition may contribute to the cellular internalization of M‐SeNPs.

**FIGURE 3:**
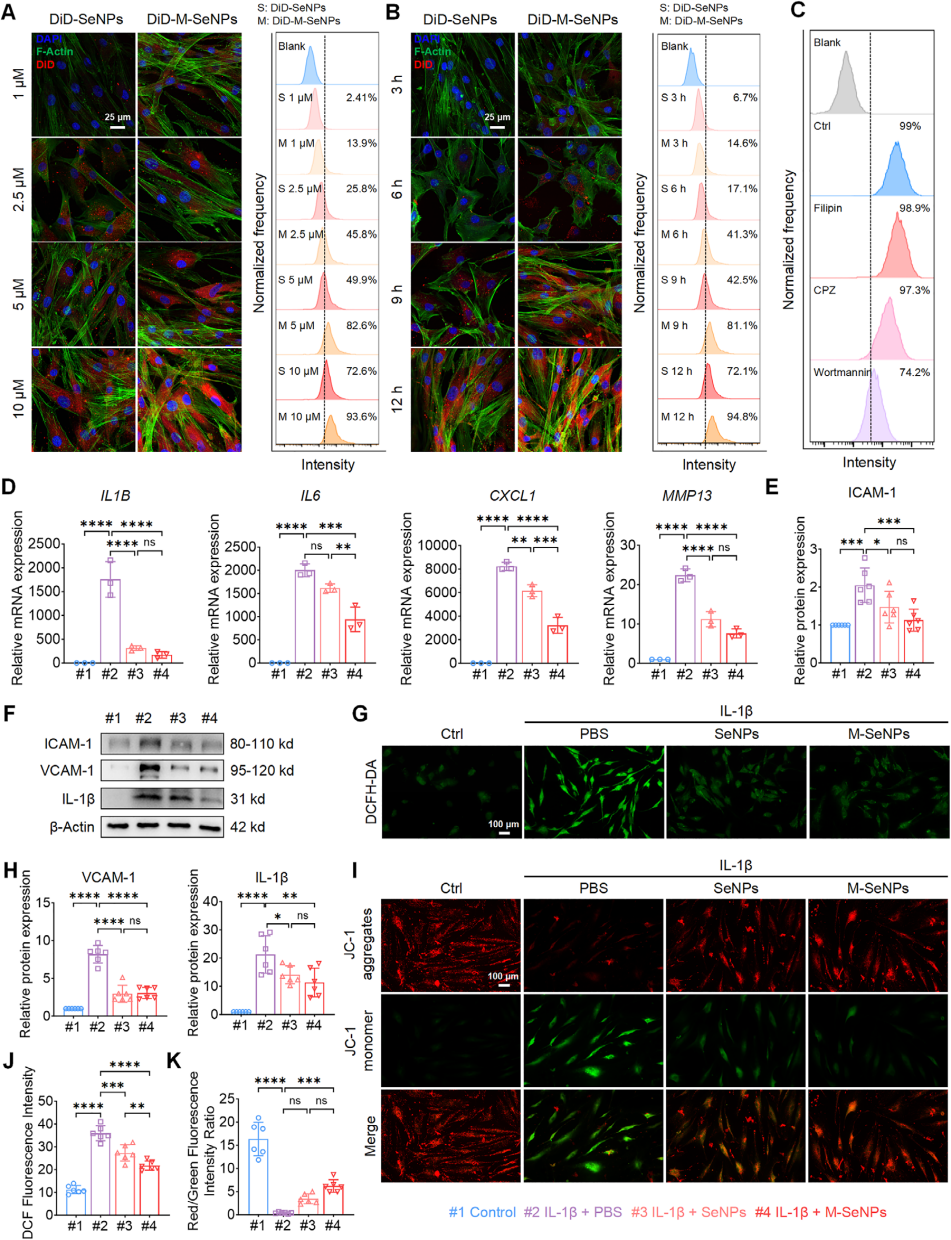
Anti‐inflammatory and anti‐oxidative effects of M‐SeNPs on FLS. (A) Representative fluorescence microscopy images and flow cytometry histograms showing concentration‐dependent uptake of DiD‐labeled SeNPs and M‐SeNPs by FLS. (B) Representative fluorescence microscopy images and flow cytometry histograms showing time‐dependent uptake of DiD‐SeNPs and DiD‐M‐SeNPs by FLS. (C) Flow cytometry histogram of cellular uptake of DiD‐M‐SeNPs in FLS pre‐treated with specific inhibitors of endocytosis: chlorpromazine, wortmannin, and Filipin. (D) Relative mRNA expression levels of *IL1B*, *IL6*, *CXCL1* and *MMP13* in FLS determined by qRT‐PCR. (E) Quantitative analysis of ICAM‐1 protein expression in FLS. (F) Representative Western blotting images of ICAM‐1, VCAM‐1, and IL‐1β expression in FLS, with β‐actin serving as a loading control. (G) Intracellular ROS levels in FLS detected by DCFH‐DA staining (n = 6). (H) Quantitative analysis of VCAM‐1 and IL‐1β protein expression in FLS. (I) Assessment of mitochondrial membrane potential (ΔΨm) in FLS using JC‐1 staining (n = 6). (J) Mean DCF fluorescence intensity reflecting intracellular ROS levels. (K) Red/green fluorescence intensity ratio (JC‐1 aggregates/monomers) indicating ΔΨm, inversely correlated with mitochondrial depolarization. #1 Control, #2 IL‐1β, #3 IL‐1β + SeNPs, and #4 IL‐1β + M‐SeNPs. Quantitative data in (D, E, H, J, K) are shown as mean ± S.D., with statistical significance determined by one‐way ANOVA. *p < 0.05, **p < 0.01, ***p < 0.001, ****p < 0.0001, ns, not significant.

To further elucidate the internalization pathways of M‐SeNPs, pharmacological inhibitors targeting caveolae/lipid raft‐mediated endocytosis (filipin), clathrin‐mediated endocytosis (chlorpromazine, CPZ), and macropinocytosis (wortmannin) were employed. Wortmannin significantly reduced M‐SeNPs uptake in both inflammatory FLS and RAW 264.7 macrophages, whereas CPZ and filipin exhibited cell‐type‐dependent effects, primarily affecting uptake in RAW264.7 macrophages (Figure 3C and Figure S9H). These results suggest that macropinocytosis represents a major internalization pathway for M‐SeNPs in inflammatory synovial cells [54, 55, 56].

Next, we assess the anti‐inflammatory activity of M‐SeNPs. In OA, activated FLS contributes to synovial inflammation by increasing the production of pro‐inflammatory cytokines (e.g., IL‐1β, IL6, IL8), chemokines (e.g., CCL2, CXCL1, CXCL2), and matrix‐degrading enzymes such as matrix metalloproteinase 13 (MMP13) [3]. Using an IL‐1β‐stimulated FLS model, we found that both SeNPs and M‐SeNPs significantly downregulated the mRNA levels of these inflammatory mediators (Figure 3D, Figure S10). Consistently, Western blotting further corroborated reduced protein levels of IL‐1β, VCAM‐1, and ICAM‐1 in IL‐1β‐stimulated FLS following SeNPs or M‐SeNPs treatment (Figure 3E, F, H). Although M‐SeNPs exhibited a tendency toward stronger inhibitory effects compared with SeNPs, differences for several inflammatory markers did not reach statistical significance, suggesting that the major advantage of mannose modification may become more apparent under the complex in vivo environment.

Since selenium is closely associated with redox regulation, we further examined oxidative stress responses. Both SeNPs and M‐SeNPs significantly reduced intracellular ROS levels in IL‐1β‐stimulated FLS (Figure 3G, J). Given that mitochondrial dysfunction often accompanies oxidative stress, we further evaluated the mitochondrial membrane potential (ΔΨm) using 5,5′,6,6′‐Tetrachloro‐1,1′,3,3′‐tetraethyl‐imidacarbocyanine iodide (JC‐1) staining. Treatment with either SeNPs or M‐SeNPs increased red/green fluorescence ratio in the inflamed cells, indicating stabilization of ΔΨm and preservation of mitochondrial integrity (Figure 3I, K).

Considering that macrophages, particularly the classically activated M1 phenotype, significantly contribute to the synovitis in OA [57, 58], we further corroborate aforementioned conclusion in LPS‐stimulated RAW 264.7 macrophages. Both SeNPs and M‐SeNPs decreased the expression of M1‐associated genes (*Il1b, Il6, Tnfa, Nos2*, and *Cd86*) and reduced the proportion of F4/80^+^CD86^+^ macrophages (Figure S11). The antioxidant and mitochondrial protective effects of selenium nanoparticles were also confirmed by DCFH‐DA and JC‐1 assays, respectively (Figure S12). These results collectively indicate that M‐SeNPs enable to attenuate inflammatory activation and oxidative stress, while stabilizing mitochondrial function in the inflamed FLS and macrophages.

### 2.4 M‐SeNPs Metabolically Reprogram Inflamed Cells via Regulating Glycolytic Signals and Selenoproteins

To define the underlying mechanisms by which M‐SeNPs suppress synovial inflammation, we performed quantitative proteomics on IL‐1β‐stimulated FLS (inflammation control) against those treated with both IL‐1β and M‐SeNPs (Figure 4A). Of the 8,868 proteins quantified, 192 were significantly upregulated and 400 were significantly downregulated following M‐SeNPs treatment (Figure 4B). As expected, the expression of key inflammatory mediators, including IL‐6, CXCL1, CCL2, VCAM‐1, and ICAM‐1, were downregulated, further corroborating our functional observations (Figure S13A).

**FIGURE 4:**
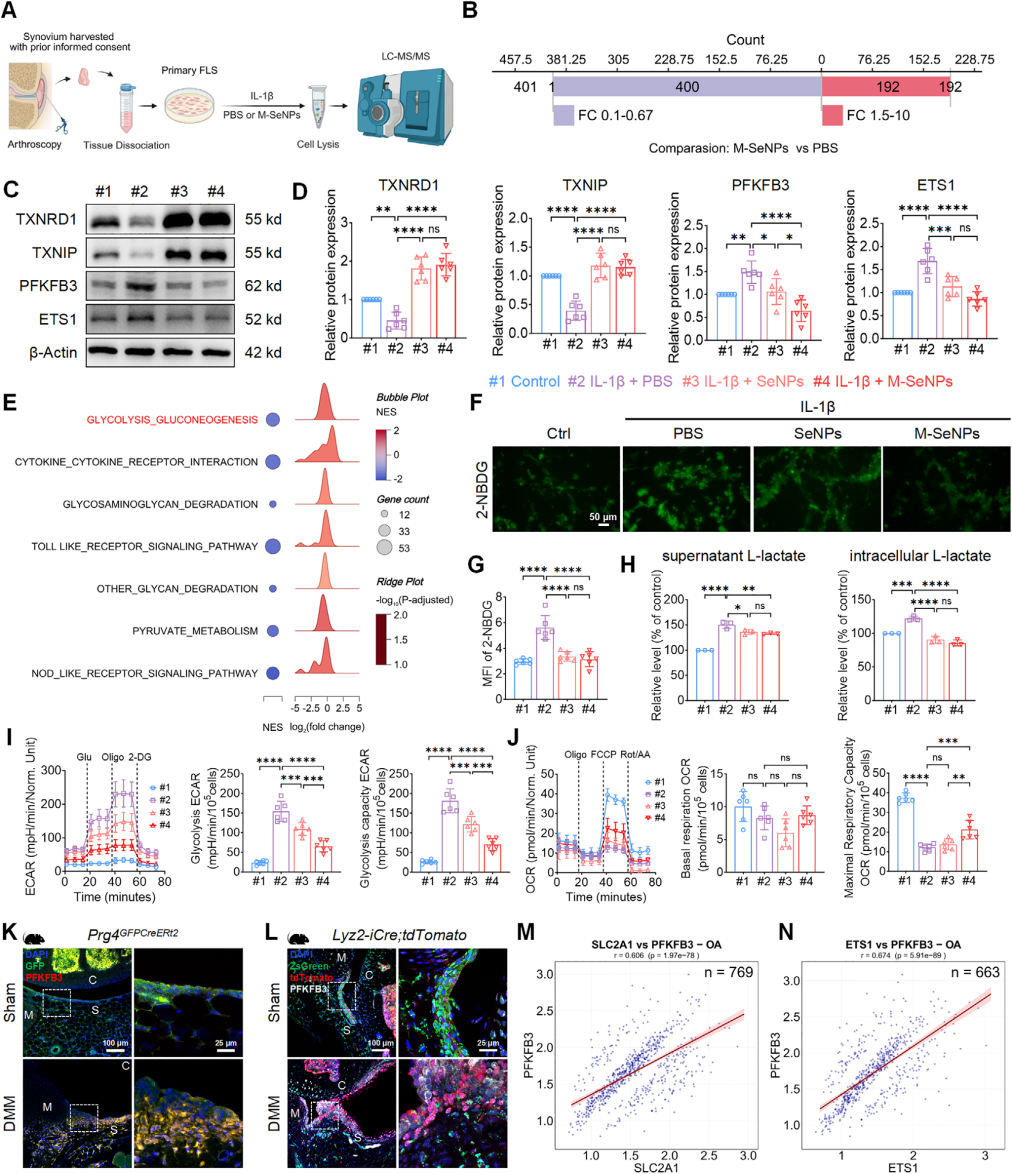
Proteomic analysis identifies metabolic and inflammatory targets of M‐SeNPs in FLS. (A) Schematic illustration of the quantitative proteomics workflow, including synovium harvest, primary FLS isolation, protein extraction and digestion, high‐resolution LC‐MS/MS analysis, and bioinformatics analyses. (Created in BioRender.) (B) Number of significantly up‐ and down‐regulated proteins between IL‐1β‐stimulated FLS with or without M‐SeNPs treatment. (C) Representative Western blotting images showing TXNRD1, TXNIP, PFKFB3, and ETS1 protein expression in FLS; β‐actin serves as a loading control. (D) Quantification of TXNRD1, TXNIP, PFKFB3, and ETS1 protein levels normalized to β‐actin. (E) Ridge plot from GSEA of differentially expressed proteins, highlighting enrichment of KEGG pathways associated with glycolysis, inflammation, and metabolic reprogramming. (F) Glucose uptake capacity of FLS detected by the 2‐NBDG fluorescent probe. (G) MFI of 2‐NBDG fluorescence reflecting cellular glucose uptake capacity. (H) Relative L‐lactate levels in culture supernatants and intracellular lysates, normalized to the control group. (I) Quantitative assessment of glycolytic flux in FLS using Seahorse XF for ECAR analysis. (J) Quantitative assessment of mitochondrial respiratory capacity in FLS using Seahorse XF for OCR analysis. (K, L) Representative immunofluorescence images showing PFKFB3 expression in GFP^+^ FLS from *Prg4^GFPCreERt2^* mice (K) and tdTomato^+^ macrophages from *Lyz2‐iCre;tdTomato* mice (L). (M, N) Scatter plots showing correlations between log‐normalized single‐cell expression of PFKFB3 and SLC2A1 (M), and PFKFB3 and ETS1 (N). Pearson correlation coefficients and corresponding *p* values are indicated. #1 Control, #2 IL‐1β, #3 IL‐1β + SeNPs, and #4 IL‐1β + M‐SeNPs. C, Cartilage; M, Meniscus; S, Synovium. Quantitative data in (D, G‐J) are shown as mean ± S.D., with statistical significance determined by one‐way ANOVA. *p < 0.05, **p < 0.01, ***p < 0.001, ****p < 0.0001, ns, not significant.

Since preliminary metabolic flux data revealed that M‐SeNPs are converted to SeCys2, a precursor for selenoprotein synthesis [59, 60], we focused on alterations in the selenoproteome. Multiple selenoproteins were upregulated after M‐SeNPs treatment. TXNRD1 showed the largest increase (> 2‐fold, *p* < 0.001), suggesting activation of thiol‐redox homeostasis, which is subsequently confirmed via Western blotting analysis (Figure 4C, D). In addition, several endoplasmic reticulum (ER)‐ and redox‐associated selenoproteins, including SELENOS [61, 62], SELENOT [63, 64, 65, 66], SELENOM, [67, 68] SELENOF, and SELENOH, were increased (Figure S13A). Gene Ontology (GO) enrichment analysis of the differentially expressed proteins further confirmed a significant enrichment of terms related to “response to oxidative stress” and “cellular redox homeostasis”.

Proteomic pathway analysis further revealed broad metabolic remodeling after M‐SeNPs treatment, including alterations in HIF‐1 signaling, TCA cycle, and pyruvate metabolism (Figure S13B). Gene Set Enrichment Analysis (GSEA) revealed a significant negative enrichment of the “Glycolysis/Gluconeogenesis” pathway in the M‐SeNPs‐treated group (Figure 4E). Functional metabolic analysis confirmed these findings, as both SeNPs and M‐SeNPs reduced extracellular acidification rate (ECAR), glucose uptake, and lactate production in IL‐1β‐stimulated FLS and LPS‐stimulated RAW264.7 macrophages, while increasing oxygen consumption rate (OCR), indicating restoration of mitochondrial metabolic activity (Figure 4F–J and Figure S14).

Consistent with these functional shifts, proteomic analysis showed a notable suppression of the pro‐glycolysis enzyme PFKFB3 and upregulation of TXNIP in M‐SeNPs‐treated group (Figure S13C). TXNIP is a known negative regulator of glucose uptake [10]; its induction by M‐SeNPs is consistent with reduced glycolytic drive. The upregulated PFKFB3 expression in OA FLS and macrophages identified by scRNA‐seq (Figure S1E) was subsequently validated via immunofluorescence in the DMM‐induced *Prg4^GFPCreERt2^* and *Lyz2‐iCre;tdTomato* mice (Figure 4K, L, and Figure S15A, B). Furthermore, PFKFB3 level significantly positively correlated with GLUT1 expression in OA FLS (r = 0.606, *p* < 0.001) (Figure 4M), suggesting a tight coupling between GLUT1‐driven glucose uptake and PFKFB3‐mediated glycolysis in OA. To further explore the transcriptional drivers underlying this metabolic reprogramming, bioinformatic analysis was performed and the transcription factor ETS1 was predicted as a potential regulator of PFKFB3 (Figure S16A). Consistently, ETS1 was abundantly expressed in the OA subpopulations (Figure S16B) while positively correlated with PFKFB3 levels (r = 0.674, *p* < 0.001) (Figure 4N). Supporting this, ETS1 abundance decreased after M‐SeNPs treatment, consistent with the trend of PFKFB3 (Figure S16C). These findings suggest that ETS1‐associated regulation of PFKFB3 may contribute to glycolytic remodeling in OA synovial cells. Finally, Western blot analysis confirmed increased TXNRD1 and TXNIP expression and reduced PFKFB3 and ETS1 expression following SeNPs or M‐SeNPs treatment (Figure 4C, D).

### 2.5 Biodistribution and Cellular Uptake of SeNPs and M‐SeNPs in Vivo

Next, we evaluated the intra‐articular retention, biodistribution, and in vivo cellular uptake of nanoparticles using a DMM‐induced mouse model of OA. DiD‐labelled SeNPs and M‐SeNPs were first prepared and then injected into the DMM‐induced OA mice joint, followed by the quantification of fluorescence intensity using an in vivo imaging system (IVIS) at predefined time points (day 0, 1, 3, 7, 10, and 14). Notably, the fluorescence signals in the free DiD and DiD‐SeNPs groups were largely diminished by day 10, while DiD‐M‐SeNPs signals persisted until approximately day 14 (Figure 5A, C, D and Figure S17). These data indicate a prolonged intra‐articular retention of M‐SeNPs relative to non‐modified SeNPs. To further assess the systemic distribution of these nanoparticles, the fluorescence was analyzed 24 h post‐injection in joint tissues and major organs. Signals were detectable not only in the local knee joints of femoral condyle (FC) and tibial plateau (TP) (Figure 5B, E), but also exhibited strong signals in the liver (rather than other major organs including heart, spleen, lung, and kidney) across groups (Figure 5F, G). This distribution pattern suggests that, following intra‐articular administration, SeNPs/M‐SeNPs are taken up locally within the knee region and subsequently undergo systemic clearance with prominent hepatic involvement, consistent with prior reports [69]. Given this, biocompatibility was also assessed, and histological examination showed no significant inflammation or tissue injury in major organs (Figure S18). In addition, routine blood counts and serum biochemistry revealed no significant differences among groups, and liver/kidney function indices remained within normal ranges (Figure S19), supporting a favorable safety profile.

**FIGURE 5:**
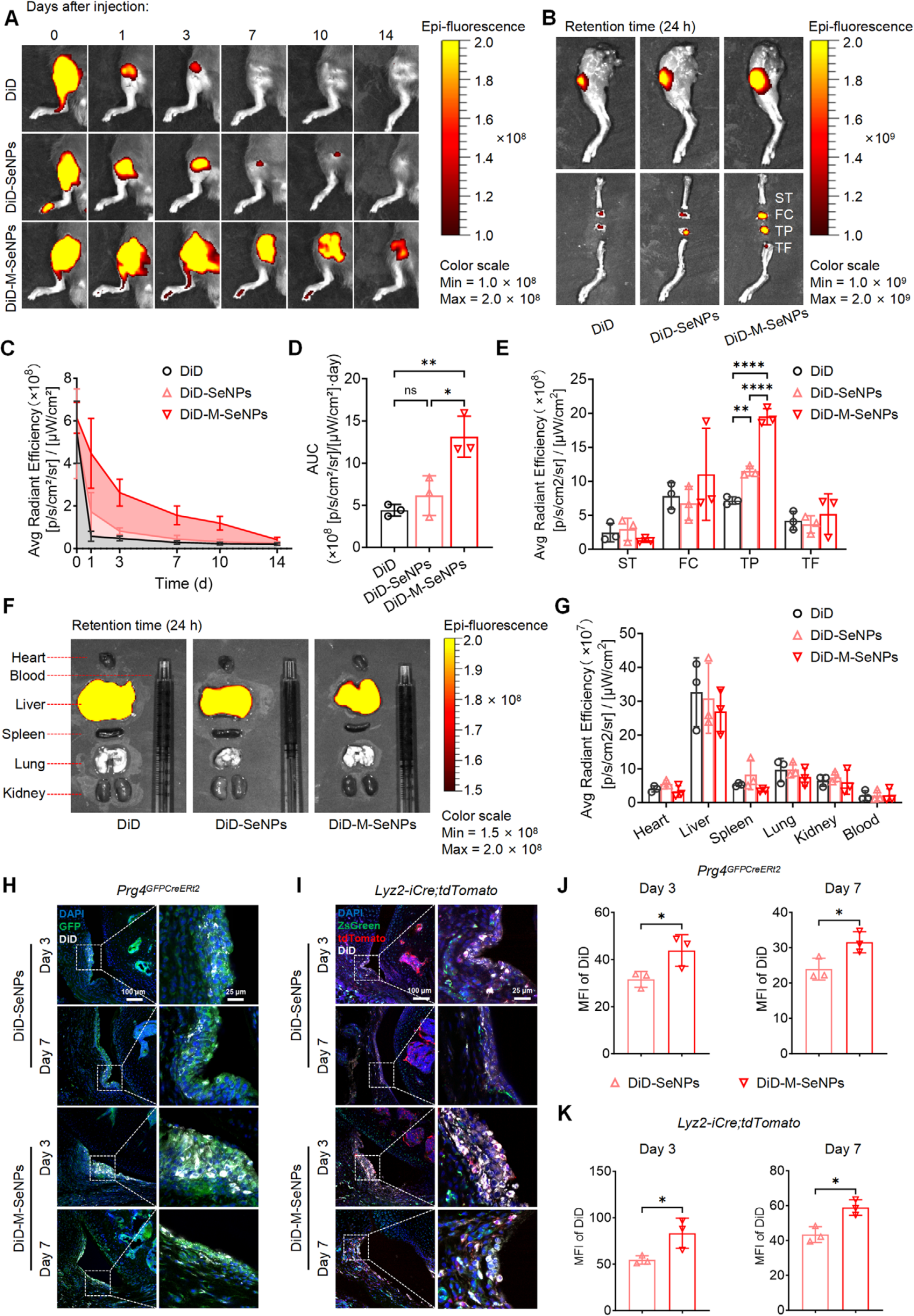
In vivo biodistribution, retention, and cellular targeting of DiD‐SeNPs and DiD‐M‐SeNPs. (A) Representative in vivo fluorescence images acquired using an IVIS imaging system, showing longitudinal monitoring of DiD fluorescence signals in the knee joint at 0, 1, 3, 7, 10, and 14 days following a single intra‐articular injection of the indicated formulations. (B) Representative ex vivo fluorescence images highlighting fluorescence distribution within distinct anatomical regions of the knee joint 24 h post‐injection. (C) Line graph showing the fluorescence intensity within articular region over the 14‐day observation period. (D) Quantification of intra‐articular residence via AUC based on the 14‐day fluorescence tracking data. (E) Average radiant efficiency in articular region at 24 h post‐intra‐articular injection. (F) Representative ex vivo fluorescence images of major organs (heart, liver, spleen, lung, and kidney) harvested 14 days post‐injection. (G) Average radiant efficiency in major organs. (H, I) Representative co‐localization immunofluorescence images showing DiD signals within GFP^+^ FLS in *Prg4^GFPCreERt2^* mice (H) and tdTomato^+^ macrophages in *Lyz2‐iCre;tdTomato* mice. (I) post‐intra‐articular injection of DiD‐SeNPs or DiD‐M‐SeNPs. (J, K) Quantification of mean DiD fluorescence intensity within GFP^+^ FLS (J) and tdTomato^+^ macrophages (K). Anatomical regions: surrounding tissue (ST), femoral condyles (FC), tibial plateau (TP), and tibiofibula (TF). n = 3. Quantitative data in (C‐E, G, J, K) are shown as mean ± S.D., with statistical significance determined by one‐way ANOVA (D, E, G) and student's t‐test (J, K). *p < 0.05, **p < 0.01, ****p < 0.0001, ns, not significant.

To determine the targeting specificity in vivo, reporter mice labeling FLS (*Prg4^GFPCreERt2^*) and macrophages (*Lyz2‐iCre;tdTomato*) were utilized. DMM‐induced mice were administered DiD‐SeNPs or DiD‐M‐SeNPs on day 7 post‐surgery. Knees were harvested at 3 and 7 day post‐injection, followed by confocal imaging at the indicated times. DMM surgery did induce marked thickening of synovium, with increased numbers of GFP^+^ FLS and tdTomato^+^ macrophages (Figure 5H‐I). Notably, intra‐articular injections of DiD‐M‐SeNPs exhibited higher co‐localization with FLS and macrophages in the DMM group at both 3 and 7 day post‐injection (Figure 5J, K). These findings indicate that mannose modification improves joint retention and enhances nanoparticle accumulation within inflammatory synovial cell populations.

### 2.6 M‐SeNPs Alleviate Pain‐Like Behaviors and Functional Impairment in OA Mice

To evaluate the therapeutic efficacy of SeNPs and M‐SeNPs in vivo, OA was induced using 8‐week‐old wild‐type mice by DMM surgery. Mice then received intra‐articular injections of an equal nanoparticle dose every 2 weeks for 12 weeks (Figure 6A). Since pain and functional decline are key clinical features of OA, then the behavioral assessments of mechanical sensitivity, motor coordination, and gait in each group were evaluated using von Frey filaments, rotarod performance and CatWalk XT gait analysis, respectively [70]. Consistent with previous reports [69, 71], DMM surgery significantly reduced paw withdraw threshold (PWT), indicating increased mechanical sensitivity in OA mice (Figure 6B), and decreased rotarod latency, consistent with impaired motor performance (Figure 6C). Gait analysis is a sensitive measure of pain and functional impairment in OA mouse models [72]. As expected, pain‐associated weight‐bearing deficits consequent gait asymmetry were evident in DMM‐induced mice. Treatment with either SeNPs or M‐SeNPs partially alleviated these gait abnormalities, as reflected by the reversal of key gait parameters: increased stride length, shortened swing time of the affected limb, improved swing speed, and a restored paw‐print area ratio between the operated (RH) and contralateral (LH) limbs (Figure 6D–H). These results collectively suggest that SeNPs or M‐SeNPs offer significant functional benefits for OA mice.

**FIGURE 6:**
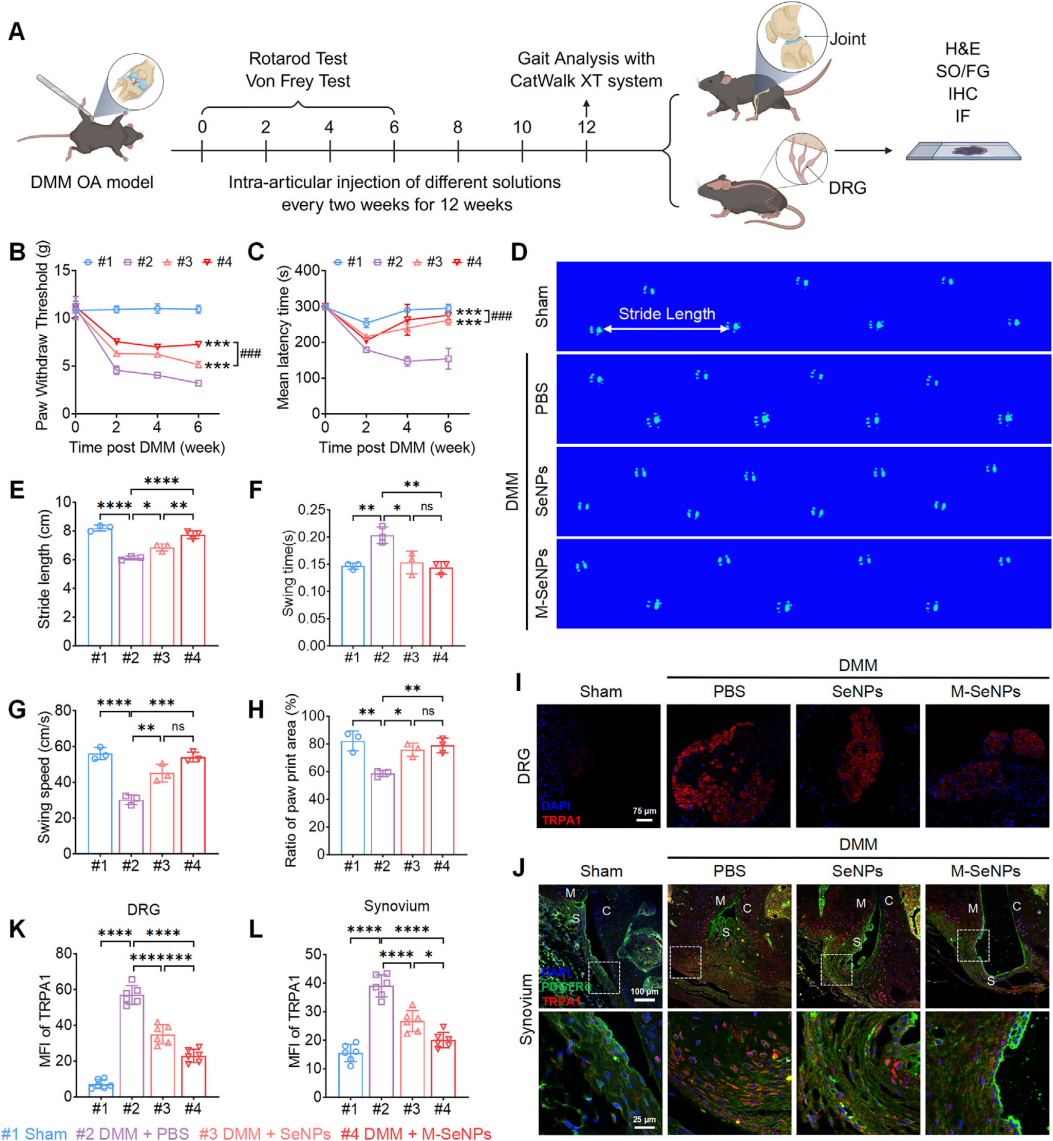
M‐SeNPs alleviate pain and functional impairment in OA mice. (A) Schematic illustration of the experimental timeline, including OA induction, intra‐articular therapeutic intervention, and sequential behavioral and histological assessments. (Created in BioRender.) (B) Time‐course analysis of mechanical hypersensitivity quantified by paw withdrawal threshold via von Frey test. (C) Assessment of motor coordination and postural balance quantified by mean latency time to fall via the Rotarod test. (D) Representative footprint captured by the CatWalk XT system, illustrating alterations in gait patterns and hindlimb weight distribution among groups (n = 3). (E) Quantification of stride length. (F) Quantification of swing time, representing the duration of the paw during the swing phase within the gait cycle. (G) Quantification of swing speed, representing average velocity of the paw during the swing phase of the gait cycle. (H) Quantification of the footprint area ratio (ipsilateral/contralateral), reflecting weight‐bearing asymmetry and compensatory off‐loading. (I, J) Representative immunofluorescence images showing TRPA1 expression and subcellular localization in DRG sections (I) and knee joint synovium (J). (K, L) Quantification of TRPA1 mean fluorescence intensity in DRG sections (K) and knee joint synovium (L) (n = 6). #1 Sham, #2 DMM + PBS, #3 DMM + SeNPs, #4 DMM + M‐SeNPs. C, Cartilage; M, Meniscus; S, Synovium. Quantitative data in (B, C, E‐H, K, L) are shown as mean ± S.D., with statistical significance determined by one‐way ANOVA. *p < 0.05, **p < 0.01, ***p < 0.001, ****p < 0.0001. ###*p* < 0.001 vs. SeNPs, ns, not significant.

We next examined transient receptor potential ankyrin 1(TRPA1), a nociception‐related ion channel implicated in OA pain and neurogenic inflammation [73]. In DMM mice, TRPA1 immunoreactivity was elevated in L3‐L4 dorsal root ganglia (DRG) innervating the affected joint [74], whereas the upregulation was significantly suppressed by SeNPs or M‐SeNPs administration (Figure 6I, K). Studies have suggested that TRPA1 activation participates in inflammatory cascades within synoviocytes, thereby exacerbating OA synovial inflammation and cartilage degeneration [75]. Herein, the TRPAl expression was also increased in the synovium upon DMM induction, whereas the use of selenium nanoparticles did inhibit the TRPAl expression within the synovium (Figure 6J, L). Together, these findings suggest that nanoparticles particularly M‐SeNPs attenuate OA‐associated pain‐like behaviors and functional impairment, at least, in part through regulating the TRPA1 expression in both peripheral sensory neurons and inflamed synovium.

### 2.7 M‐SeNPs Retard Osteoarthritis Progression via Regulation of Glycolytic Signals

To evaluate the therapeutic efficacy of selenium nanoparticles in OA, we performed histological and immunofluorescence analysis of the whole knee joints. H&E staining showed that DMM surgery induced marked synovial hyperplasia, inflammatory cell infiltration, and increased synovitis scores, whereas SeNPs and M‐SeNPs treatment significantly alleviated these pathological changes (Figure 7A, B). Safranin O/Fast Green staining further demonstrated that SeNPs and M‐SeNPs reduced proteoglycan loss and preserved cartilage integrity, as indicated by decreased OARSI scores (Figure 7C, D). Given that MMP13 serves as a core biomarker of cartilage catabolism and simultaneously reflects the severity of synovial inflammation [76, 77], we evaluated its expression via immunohistochemistry. Our analysis revealed that the expression of MMP13 in both synovium and articular cartilage was significantly downregulated following treatment, highlighting the efficacy of SeNPs or M‐SeNPs in concurrently mitigating synovial inflammation and preserving the cartilage matrix. (Figure 7E‐G).

**FIGURE 7:**
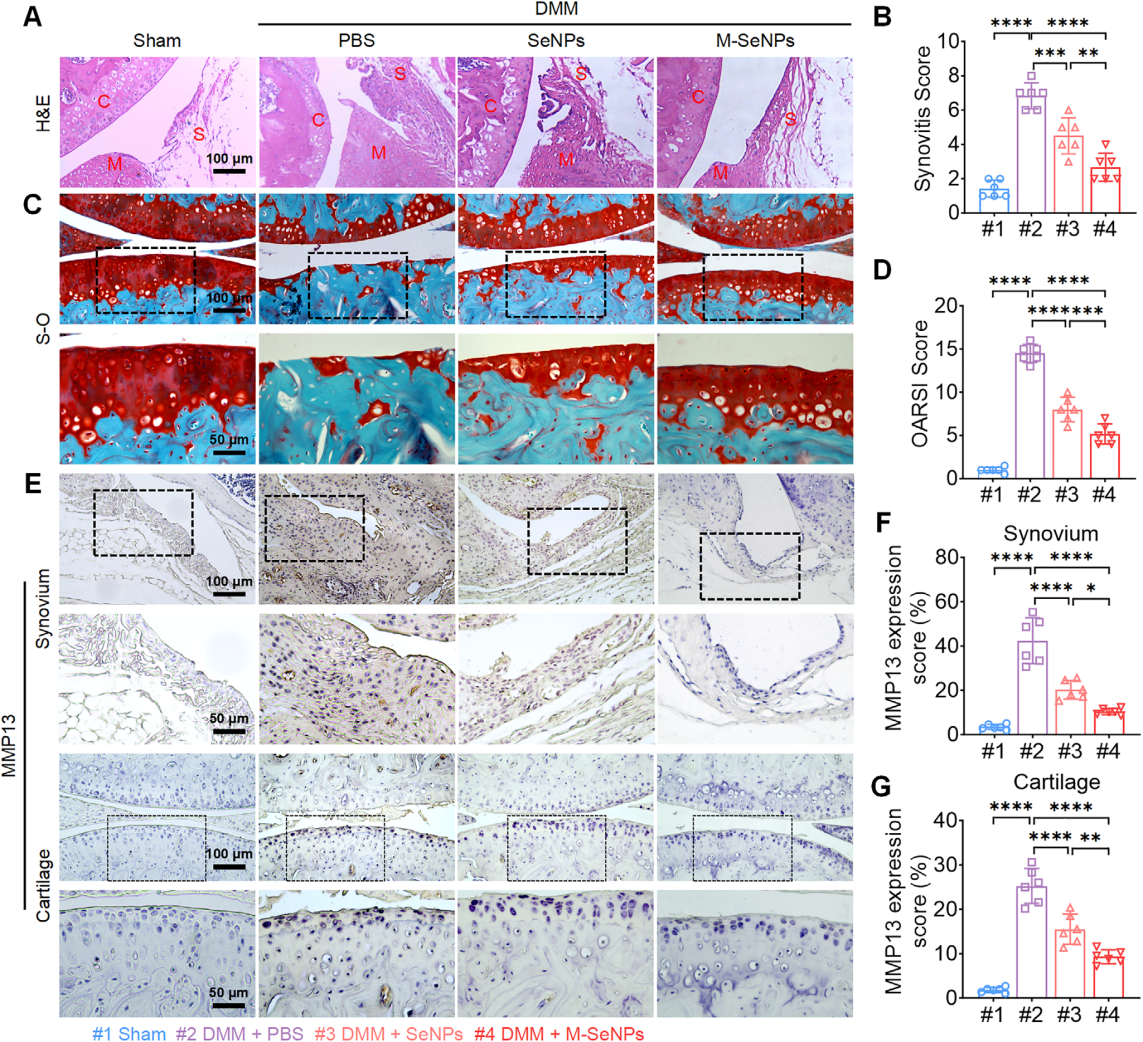
M‐SeNPs alleviate cartilage degeneration and OA progression. (A) Representative H&E‐stained synovial tissues, revealing synovial lining hyperplasia, inflammatory cell infiltration, and stromal fibrosis. (B) Quantitative synovitis scores derived from H&E staining, based on synovial lining thickness, inflammatory cell infiltration, and vascular proliferation. (C) Representative Safranin O/Fast Green‐stained articular cartilage sections showing proteoglycan content (red) and collagen‐rich matrix structure (green). (D) OARSI scores evaluating the extent of cartilage degeneration from Safranin O/Fast Green staining. (E) Representative immunohistochemical staining for MMP13 (brown) in synovium (upper panels) and articular cartilage (lower panels). (F, G) Quantification of MMP13‐positive area within synovium (F) and articular cartilage (G) normalized to total tissue area. #1 Sham, #2 DMM + PBS, #3 DMM + SeNPs, #4 DMM + M‐SeNPs. C, Cartilage; M, Meniscus; S, Synovium. Quantitative data in (B, D, F, G) are shown as mean ± S.D., with statistical significance determined by one‐way ANOVA. *p < 0.05, **p < 0.01, ***p < 0.001, ****p < 0.0001.

To corroborate the above in vitro observations, we examined the synovial macrophage polarization and key redox/metabolic markers. Immunofluorescence staining showed a siginificant increase of F4/80 (pan‐macrophage marker) and iNOS (M1 macrophage marker) co‐positive cells in the DMM synovium, whereas SeNPs/M‐SeNPs reduced the accumulation of F4/80^+^iNOS^+^ M1‐like macrophages in the DMM synovium (Figure 8A‐C). Meanwhile, M‐SeNPs treatment significantly increased the proportion of F4/80^+^CD206^+^ macrophages in the synovium of DMM mice, indicating an enhanced anti‐inflammatory M2‐like macrophage phenotype. (Figure S20) [78, 79]. In addition, both SeNPs and M‐SeNPs were found to partially restore the TXNRD1 (Figure 8D, E)and TXNIP (Figure 8F, G) expression in the GLUT1^high^ synoviocytes of the DMM group, compared to the sham group. Given the potential fundamental role of the ETS1/PFKFB3 axis in synoviocytes, we also assessed its expression in vivo. In line with our cellular findings above, DMM induction elevated the expression of ETS1 and PFKFB3, a trend that was reversed following M‐SeNPs treatment (Figure 8H–J). Collectively, these results indicated that M‐SeNPs mitigate OA synovial inflammation and cartilage degeneration via targeting the GLUT1^high^ synoviocytes, partially through association with redox reinforcement and suppression of glycolytic metabolism within synovium.

**FIGURE 8:**
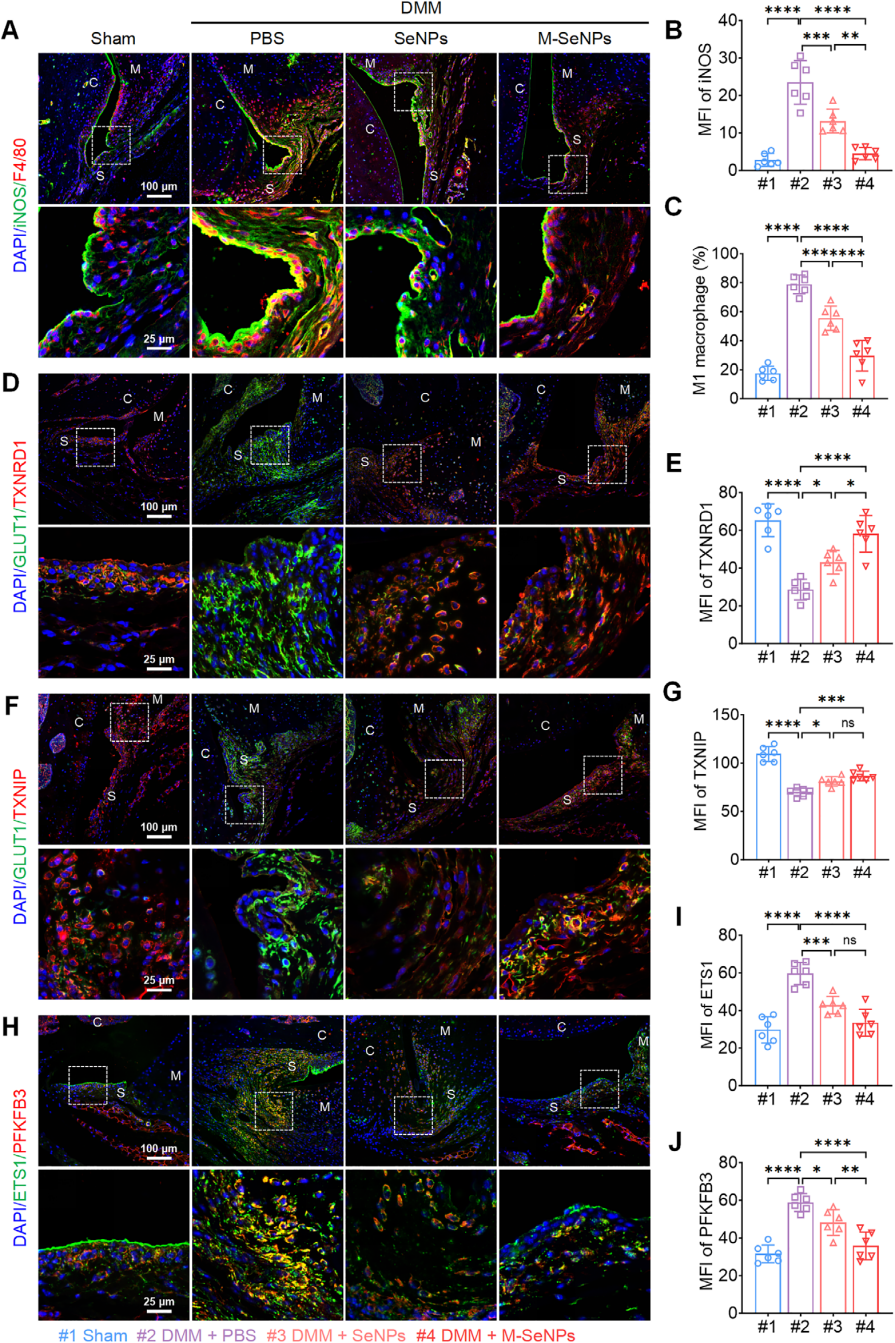
M‐SeNPs attenuate inflammation and restore tissue homeostasis by targeting specific proteins within key regulatory pathways. (A) Representative immunofluorescence co‐staining of iNOS and F4/80 in knee joint synovium. Co‐localized iNOS^+^F4/80^+^ signals indicate pro‐inflammatory M1 macrophages. (B) Quantification of MFI of iNOS in synovium. (C) Quantitative analysis of M1 macrophage proportion in synovium. M1 macrophages were defined as iNOS^+^F4/80^+^ cells, and percentages were calculated as the number of iNOS^+^ cells relative to total F4/80^+^ macrophages across multiple high‐power fields. (D) Representative immunofluorescence co‐staining of GLUT1 and TXNRD1 in synovium. (E) Quantification of TXNRD1 MFI in synovium. (F) Representative immunofluorescence co‐staining of GLUT1 and TXNIP in synovium. (G) Quantification of TXNIP MFI in synovium. (H) Representative immunofluorescence co‐staining of ETS1 and PFKFB3 in synovium. (I‐J) Quantification of ETS1 (I) and PFKFB3 (J) MFI in synovium. #1 Sham, #2 DMM + PBS, #3 DMM + SeNPs, #4 DMM + M‐SeNPs. C, Cartilage; M, Meniscus; S, Synovium. Quantitative data in (B, C, E, G, I, J) are shown as mean ± S.D., with statistical significance determined by one‐way ANOVA. *p < 0.05, **p < 0.01, ***p < 0.001, ****p < 0.0001, ns, not significant.

## 3 Discussion

OA is a degenerative joint disease characterized by progressive cartilage loss and persistent synovial inflammation, representing a leading cause of chronic pain and disability worldwide [80]. Although disease‐modifying strategies are actively being explored, many are limited by inadequate targeting of the core pathological mechanisms that drive OA. Emerging evidence has suggested that synovial inflammation is a central driver of OA pathogenesis [5, 81], fueled by the synergistic dysregulation of glycolytic metabolism [12, 82] and oxidative stress [83]. For instance, activated synoviocytes undergo metabolic reprogramming toward glycolysis, resulting in excessive lactate accumulation, elevated ROS production, and enhanced pro‐inflammatory signaling, which drive joint tissue damage and degeneration [6, 9, 84].

GLUT1 serves as a crucial regulator of glycolytic flux by modulating cellular glucose uptake, and its aberrant activation has been increasingly implicated in the progression of OA [12, 85]. Our previous studies have demonstrated that enhanced GLUT1‐dependent glycolysis in synovial fibroblasts is closely associated with disease severity, highlighting GLUT1 as a metabolic gatekeeper linking energy metabolism to synovial inflammation. ScRNA‐seq analysis revealed that both FLS and macrophages in OA exhibit a pronounced transcriptional signature of glycolysis, characterized by marked upregulation of SLC2A1. Further analysis verified that SLC2A1^+^ subsets exhibit robust pro‐inflammatory, pro‐fibrotic, and catabolic features. Pseudotime analysis suggested that high SLC2A1 expression is associated with a terminal pathological state of OA FLS under inflammatory conditions, potential contributing to the pregression of OA pathology. Integrative biochemical analysis of clinical samples further revealed disruption of the synovial redox system, particularly involving impaired selenoproteins expression. These findings were validated in vivo using fluorescence‐traced, cell type‐specific reporter mice, collectively substantiating that coupled metabolic reprogramming and redox imbalance constitute a central mechanistic axis driving synovial inflammation in OA (Figure 1).

Based on the GLUT1‐overexpressing metabolic phenotype of inflammatory synoviocytes, we developed M‐SeNPs as an intra‐articular nanotherapeutic strategy to enhance the inflamed cellular uptake. Subsequent in vivo studies, using IVIS imaging and fluorescence reporter mice demonstrated that mannose modification markedly enhanced intra‐articular retention and preferential targeting of pathogenic synovial cell populations, while improving delivery efficiency and minimizing extra‐articular distribution (Figures 2, and 5). Following cellular internalization, M‐SeNPs exerted a synergistic dual‐regulatory effect. M‐SeNPs function as a sustained selenium supply platform, metabolically converted into the bioactive form SeCys_2_. Proteomic and functional analyses identified that M‐SeNPs restore a selenoprotein associated redox‐regulatory network, consistent with reduced ROS accumulation and recovery of mitochondrial membrane potential. Previous studies have shown that antioxidant selenoproteins (TXNRDs and GPXs) are reduced in OA cartilage, while selenium supplementation improves mitochondrial function and alleviates joint degeneration [30]. Among the differentially expressed selenoproteins identified in our analyses, TXNRD1 exhibited a prominent responses to M‐SeNPs treatment and was selected for further validation. Previous studies suggest that TXNRD1 contributes to intracellular redox homeostasis and limits oxidative signaling under inflammatory conditions [60, 85]. In this study, restoration of TXNRD1 expression was accompanied by reduced oxidative stress and inflammatory activation, suggesting that TXNRD1 may contribute to the protective effects of M‐SeNPs. However, the current data do not establish a direct causal role for TXNRD1, and further loss‐of‐function and rescue experiments are required to determine its mechanistic involvement in the anti‐inflammatory effects of M‐SeNPs. Importantly, oxidative stress in OA synovitis acts as an upstream amplifier, reinforcing cytokine production, inflammation activation, and catabolic mediator expression beyond its role as a downstream consequence [86]. Thus, these results suggest that M‐SeNPs mitigate synovial inflammation by reconstituting endogenous, selenoprotein‐mediated redox buffering rather than by providing a single exogenous antioxidant (Figures 3 and 4).

Concomitantly, M‐SeNPs suppressed aberrant glycolytic metabolism. Proteomic and functional metabolic analyses revealed that M‐SeNPs exposure reduced glycolytic flux alongside enhanced mitochondrial respiration, as evidenced by decreased ECAR, glucose uptake capacity, and lactate production, while increasing OCR (Figure 4), indicating a bioenergetic shift away from glycolysis. Glycolysis sustains both the bioenergetics and the pro‐inflammatory signaling of M1 macrophages and activated FLS, which drive OA progression. We have previously demonstrated that TXNIP is downregulated in OA [10]. In the current study, the expression of TXNIP was upregulated by M‐SeNPs. On the other hand, we consistently observed coordinated upregulation of ETS1 and PFKFB3 in OA, both of which were suppressed following M‐SeNPs treatment. Based on the previous research findings, PFKFB3 potently promotes glycolytic flux [21, 87, 88, 89], and is transcriptionally activated by ETS1 [90]. The ETS1‐PFKFB3 axis may be involved in the metabolic and anti‐inflammatory effects of M‐SeNPs. However, our findings only indicate an association between ETS1 and PFKFB3 expression in OA synovial tissues, and further studies are required to validate their direct regulatory relationship.

Importantly, these molecular and cellular improvements translated directly into significant amelioration of OA. M‐SeNPs are well tolerated upon repeated intra‐articular administration and significantly alleviate pain‐related behaviors, improve joint function, and reduce expression of the nociceptive channel TRPA1 (Figure 6). The N‐terminus of the TRPA1 protein contains 14–18 ankyrin repeats, which constitute the core sensing region for ROS/electrophiles [91]. Previous studies have suggested that various reactive oxygen and nitrogen species, including hydrogen peroxide (H_2_O_2_), hydroxyl radicals (·OH), and nitric oxide (NO), as well as endogenous lipid peroxides such as 4‐hydroxynonenal (4‐HNE), can activate TRPA1 channels through modification of key cysteine residues. TRPA1 activation subsequently serves as an important upstream signaling event in the pathogenesis of multiple chronic pain conditions, including neuropathic pain, inflammatory pain, and chemotherapy‐induced pain [92, 93, 94]. Although it remains to be determined whether TRPA1 suppression represents a direct effect of M‐SeNPs or occurs secondary to reduced synovial inflammation and metabolic stress, improvement in the pain behaviors suggests that reprogramming the synovial inflammatory‐metabolic state can modulate peripheral nociceptive sensitization. Histopathological and immunofluorescence analysis further demonstrate that M‐SeNPs significantly alleviate synovitis, preserve cartilage integrity, and reprogram macrophage polarization from pro‐inflammatory M1 toward anti‐inflammatory M2 phenotypes (Figures 7 and 8).

Collectively, these findings establish superior chondroprotection effects of M‐SeNPs in an OA mouse mode, at least in part, through reconstructing glycolysis and redox homeostasis in the GLUT1^high^ synoviocytes. In summary, these findings demonstrate that targeting synovial metabolism via mannose‐modified nanoparticles is a promising new avenue for OA therapy.

## 4 Conclusion

In summary, our scRNA‐seq analysis revealed enhanced glycolytic activation in OA synovium, characterized by elevated GLUT1 expression and impaired selenium‐dependent antioxidant defenses, which may contribute to persistent synovial inflammation. Based on this pathological feature, we developed an intra‐articular nanomedicine strategy using M‐SeNPs to preferentially target metabolic activeated synovial cells. M‐SeNPs subsequently modulate synovial metabolic dysregulation and redox imbalance, thereby attenuating inflammatory amplification and cartilage degradation. Collectively, this study provides a mechanistic rationale for the development of selenium‐based nanotherapeutics as disease‐modifying interventions for osteoarthritis. (Figure 9, Created in https://BioRender.com).

**FIGURE 9:**
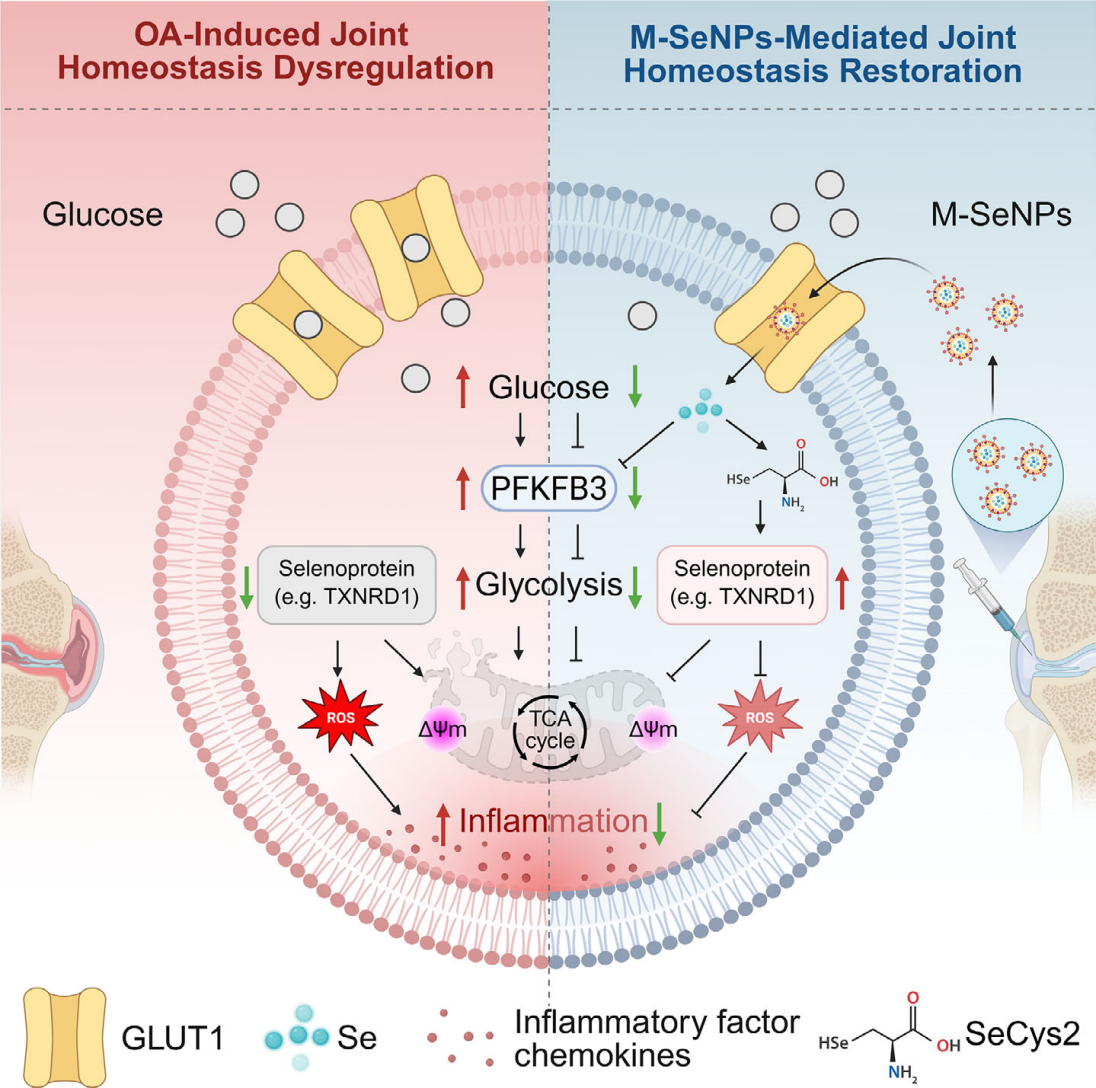
A schematic diagram of reprogramming synovial glycolysis and redox homeostasis with M‐SeNPs to ameliorate OA. In osteoarthritic synovium, FLS and macrophages are characterized by elevated GLUT1 expression and a deficiency in selenoproteins, leading to glycolysis aggravation, ROS accumulation, mitochondrial dysfunction, and sustained inflammatory activation. To restore joint homeostasis, intra‐articular delivery of M‐SeNPs preferentially targets hypermetabolic synoviocytes via GLUT1‐dependent uptake. Serving as a bioavailable selenium source, M‐SeNPs restore redox homeostasis via upregulation of selenoproteins (e.g., TXNRD1). Simultaneously, M‐SeNPs reduced the elevated PFKFB3 expression and attenuated glycolytic activity in inflamed cells. Ultimately, M‐SeNPs retard osteoarthritis progression and alleviate pain‐like behaviors and functional impairment in mice through the regulation of antioxidant and glycolytic signals.

## 5 Experimental Methods

### 5.1 Ethics Statement

Human synovial tissues were obtained from the suprapatellar synovial lining region of the knee joint from patients with OA or isolated anterior cruciate ligament (ACL) injury without radiographic evidence of OA. Non‐hemorrhagic synovial fluid samples were extracted from the joint cavity. The study protocol was reviewed and approved by the Ethics Committee of the First Affiliated Hospital of Jinan University (Ethical approval number: KY‐2021‐064, KY‐2021‐022, [2019]‐KY‐001). All patients signed an informed consent form approved by the local institutional review board. All human samples were anonymized and processed in a de‐identified manner. All animal procedures conformed to the “Guide for the Care and Use of Laboratory Animals” of the National Institute of Health in China.

All animal procedures were reviewed and approved by the Institutional Animal Care and Use Committee (IACUC) of Jinan University (Approval number: IACUC‐20241022‐05)

### 5.2 ScRNA‐seq

ScRNA‐seq was performed and analyzed as described in the previous study with the following details. In brief, synovial tissues were digested using a mixture of collagenase I/II/IV and neutral protease (total 3 mg mL^−^^1^, ratio 1:2:2:1) with 10 µg mL^−^^1^ DNase I at 37°C for 40 min. After termination with 10% FBS‐DMEM, filtration (70 µm), centrifugation (500 g, 5 min, 4°C), and red blood cell lysis, single‐cell suspensions were obtained. Approximately 20,000 viable cells per sample were loaded onto a 10x Genomics Chromium Controller (v3 chemistry) targeting ∼10,000 cells per library. Libraries were sequenced on an Illumina HiSeq platform (2 × 150 bp paired‐end) at ∼400 million reads per sample. Raw data were processed with Cell Ranger v6.0.2 (GRCh38‐2020‐A). Downstream analyses including quality control, clustering (Seurat v4), batch correction (Harmony v1.0), UMAP visualization, and differential expression testing (Wilcoxon rank‐sum test) were performed as previously detailed [10]. Gene Ontology (GO) and KEGG enrichment analyses were conducted using clusterProfiler. Gene Set Enrichment Analysis (GSEA) was performed using the MSigDB database. Gene set activity scoring was carried out with AUCell. Heatmaps and other custom visualizations were generated using ggplot2 and pheatmap. Cellular differentiation potency was analyzed via the CytoTRACE package.

### 5.3 Proteomic Analysis

Proteomic analysis was conducted using the Oscar DIA quantitative proteomics platform (Shanghai Applied Protein Technology, China) and performed as described in the previous study [60]. FLS were stimulated with 10 ng mL^−^^1^ IL‐1β ± M‐SeNPs for 24 h (n = 3 per group). Proteins were extracted using SDT lysis buffer (4% SDS, 100 mM Tris‐HCl pH 7.6, 0.1 M DTT), and protein concentration was measured using the BCA method. Proteins were digested using the filter‐aided sample preparation (FASP) method and analyzed by mass spectrometry with data‐independent acquisition (DIA) (400 ng peptides per sample). Differentially expressed proteins were defined as those with fold change > 1.5 (up‐regulated) or < 0.67 (down‐regulated) and *p* < 0.05 (t‐test). Gene Ontology (GO) functional annotation was performed using Blast2GO software. Kyoto Encyclopedia of Genes and Genomes (KEGG) pathway enrichment analysis was conducted using Fisher's exact test. Gene Set Enrichment Analysis (GSEA) was performed to identify biological pathways significantly enriched among the differentially expressed proteins. A significance threshold of adjusted *p* < 0.05 was applied for all enrichment analyses.

### 5.4 Cell Culture

RAW 264.7 macrophages were obtained from the American Type Culture Collection (ATCC). Primary human FLS were isolated using previously established methods, following the acquisition of written informed consent [95]. The cells were cultured in DMEM (Gibco) supplemented with 10% fetal bovine serum (FBS), 100 U mL^−1^ penicillin, and 100 µg mL^−1^ streptomycin at 37 °C in 5% CO_2_. RAW 264.7 macrophages at passages 10–15 and FLS at passages 3–6 were used for the experiments.

### 5.5 Preparation of SeNPs and M‐SeNPs

DSPE‐PEG‐SeNPs (SeNPs) and DiD‐labeled SeNPs (DiD‐SeNPs) were prepared at room temperature. Briefly, 20 mg of DSPE‐PEG, with or without 2 mg of DiD, was dissolved in 0.2 mL of ethanol and added to 10 mL of water. Next, 0.1 mL of sodium selenite (100 mM) and 0.1 mL of ascorbic acid (400 mM) were added dropwise under stirring. After stirring for 12 h, the resulting nanoparticles were purified by dialysis (MWCO 50 kDa) against a 100‐fold volume of deionized water for 3 days. To prepare mannose‐modified SeNPs (M‐SeNPs) or DiD‐labeled M‐SeNPs (DiD‐M‐SeNPs), 5 mg of SeNPs or DiD‐SeNPs was dispersed in 10 mL of water, followed by the addition of 0.1 mmol each of DMAP and DCC and 2 mg of mannose. After stirring for 2 h, the products were purified by dialysis (MWCO 50 kDa) against deionized water for 3 days.

### 5.6 Characterization of SeNPs and M‐SeNPs

The morphology and elemental composition of SeNPs and M‐SeNPs were characterized using high‐resolution transmission electron microscopy (HR‐TEM, JEOL 2010, 200 kV) coupled with energy dispersive X‐ray spectroscopy (EDS, X‐MaxTEM). Particle size and zeta potential of Mannose‐DSPE‐PEG‐SeNPs were determined with Zetasizer Nano ZS analyzer (Malvern Instruments Ltd). The chemical structure of M‐SeNPs was analyzed by UV–Vis‐NIR spectroscopy (Cary 5000 spectrophotometer) and Fourier‐transform infrared spectroscopy (FTIR, Nicolet iS10, Thermo Fisher Scientific). A cell imaging reader (BioTek Cytation 5) was employed for in vitro fluorescence assays.

Selenium metabolic flux was assessed in cells treated with the experimental materials over a time course. Briefly, 1 × 10^6^ primary human FLS were harvested after the designated treatment periods. Cells were lysed using a sequential enzymatic digestion with proteinase K and trypsin, followed by overnight incubation at 37°C with gentle shaking. The lysates were then further disrupted by sonication and centrifuged at 12,000 rpm to collect the supernatant. Selenium‐containing metabolites in the supernatant were separated and quantified using high‐performance liquid chromatography coupled with inductively coupled plasma mass spectrometry (HPLC‐ICP‐MS).

### 5.7 Cellular Uptake and Endocytosis Pathway Analysis

RAW 264.7 macrophages or FLS were incubated with DiD‐SeNPs or DiD‐M‐SeNPs in complete medium. Cells were washed with PBS, fixed with 4% paraformaldehyde, permeabilized with 0.1% Triton X‐100, and stained with Actin‐Tracker Green‐488 (1:200, Beyotime) and DAPI (1:1000, CST). Cellular internalization was observed via a Leica STELLARIS 5 confocal microscope and quantitatively assessed by flow cytometry (BD LSRFortessa) with DiD fluorescence measured in the APC channel. Data were analyzed using FlowJo v10.8.1.

### 5.8 Western Blotting Analysis

Western blotting analysis was conducted following established protocols. Protein concentration was determined by BCA assay. Equal amounts of protein (20 µg) were separated by SDS‐PAGE and then electrotransferred onto PVDF membranes. After blocking, the membranes were incubated with specific primary antibodies (refer to Table S1 for details) at 1:1000 dilution overnight at 4°C. Following secondary antibody incubation (HRP‐conjugated anti‐rabbit/mouse IgG, 1:2000 dilution, 1 h, at room temperature), protein bands were visualized using Clarity Western ECL substrate (Bio‐Rad) and imaged with a chemiluminescence imaging system (Tanon 5200, Tanon, China). β‐Actin was used as a loading control. Band intensities were quantified using ImageJ.

### 5.9 RNA Extraction and Quantitative Real‐Time PCR (qRT‐PCR)

Total RNA was extracted from snap‐frozen cell pellets using TRIzol reagent (Invitrogen) and purified by chloroform‐isopropanol precipitation. RNA purity (A260/A280 1.8–2.0) and concentration were assessed using a NanoDrop 2000 spectrophotometer. 1 µg of RNA was reverse‐transcribed into cDNA with TransScript First‐Strand cDNA Synthesis SuperMix kit (TransGen, AQ211) according to the manufacturer's instructions. cDNA was then diluted 1:10 with nuclease‐free water, and 2 µL was used as template for qRT‐PCR. Reactions (20 µL final volume) contained 10 µL PerfectStart Green qPCR SuperMix (TransGen, AQ601‐01‐V2), gene‐specific primers (1 µM each), and cDNA template. Each sample was run in triplicate and relative mRNA expression of target genes was calculated using the 2^−ΔΔCt^ method with β‐Actin as the endogenous control. Primer sequences are listed in Table S2.

### 5.10 Immunohistochemistry

Tissue sections were deparaffinized in xylene and rehydrated through a graded ethanol series (100% to 70%). Antigen retrieval was performed in preheated Tris‐EDTA solution (pH 9.0) at 85°C for 25 min. Endogenous peroxidase activity was quenched with 3% H_2_O_2_ for 20 min at room temperature, followed by 5% BSA blocking for 30 min. Sections were incubated overnight at 4°C with primary antibody (see Table S1 for details) at 1:200 dilution. After washing with PBS, sections were incubated with HRP‐conjugated secondary antibodies at 1:500 dilution for 1 h at room temperature. DAB chromogen was applied until optimal signal developed. Sections were then counterstained with hematoxylin, dehydrated, cleared in xylene, and mounted with a permanent mounting medium.

### 5.11 Immunofluorescence

The tissue processing steps prior to secondary antibody incubation were identical to those described for immunohistochemistry. Following PBS washes, Alexa Fluor‐conjugated secondary antibodies at 1:500 dilution were applied for 1 h at room temperature. Nuclei were counterstained with DAPI for 5 min. Finally, sections were mounted with a suitable antifade mounting medium and imaged using a laser scanning confocal microscope. The information of antibodies was described in Table S1.

For multi‐color immunofluorescence staining, the TSAPlus Fluorescence Triple‐Labeling Four‐Color Kit (Servicebio, #G1236) was used according to the manufacturer's instructions. Briefly, after antigen retrieval, blocking, and overnight incubation with primary antibodies, sections were sequentially incubated with HRP‐conjugated secondary antibody and TSA‐fluorescein substrates for each target. Nuclei were counterstained with DAPI. Multi‐color images were captured using a confocal microscope.

### 5.12 Mouse Knee OA Model and Intra‐Articular Drug Injection

8‐week‐old male C57BL/6J mice were anesthetized with pentobarbital sodium (50 mg kg^−^^1^ body weight, i.p.) and an OA model was established by performing DMM surgery on the right knee joint. Successful DMM surgery was confirmed intra‐operatively by complete transection of the medial meniscotibial ligament (MMTL) using micro‐scissors after skin incision and medial parapatellar arthrotomy, as evidenced by visibly increased mobility of the medial meniscus. The joint capsule and skin were then closed with 6‐0 nylon sutures. The sham surgery group underwent only joint capsule incision. Mice were randomly assigned to 4 groups (n = 6 each): sham + PBS, DMM + PBS, DMM + SeNPs, and DMM + M‐SeNPs. Intra‐articular injections (20 µL of SeNPs or M‐SeNPs in PBS, or PBS alone) were administered via the patellar ligament route starting at day 7 post‐surgery, once every two weeks for 12 weeks. Mice were euthanized 48 h after the final injection, and knee joints and DRG (L3‐4) were collected for histological analysis.

### 5.13 Establishment of Fluorescent Tracking Mice

For inducible and specific labeling of FLS, *Prg4^GFPCreERt2^* (*Prg4^1(GFP/Cre/ERT2) Abl/J^*, 022757) mice were kindly presented to us by Professor Ma Xiaoyuan from Qilu Hospital of Shandong University. In this model, the proteoglycan 4 (PRG4, also known as Lubricin) gene promoter drives the expression of a fusion protein consisting of Cre recombinase and a mutated estrogen receptor ligand‐binding domain (ERt2). GFP expression is permanently activated exclusively in cells that express PRG4.

To track the lineage of myeloid‐derived cells and their dynamic behaviors in vivo, a novel double transgenic Cre‐loxP lineage tracing model by crossing two established mouse strains purchased from GemPharmatech (Nanjing, China) was generated: the *B6‐Lyz2‐iCre* (*C57BL/6JGpt‐Lyz2^em1Cin(iCre)/Gpt^*) driver line and the *B6‐G/R* (*B6/JGpt‐H11^em1Cin(CAG‐LoxP‐ZsGreen‐Stop‐LoxP‐tdTomato)/Gpt^*) reporter line. *B6‐Lyz2‐iCre* mice express codon‐optimized Cre recombinase (iCre) under the control of the endogenous Lyz2 promoter, enabling robust and specific gene recombination in myeloid lineages, particularly macrophages. The *B6‐G/R* mice harbor a CAG‐driven dual‐fluorescence reporter at the H11 locus, ubiquitously expressing ZsGreen for cellular visualization until Cre‐mediated recombination triggers a permanent switch to tdTomato. Crossing *B6‐Lyz2‐iCre* and *B6‐G/R* mice yields double transgenic offspring *Lyz2‐iCre;tdTomato* where iCre‐mediated excision of the ZsGreen‐STOP cassette permanently switches fluorescence from green to red in Lyz2‐active myeloid lineages and their progeny.

The genotypes of the mice were identified according to the manufacturer's instructions (Selleck, Mouse Direct PCR Kit, USA). In brief, DNA was extracted from mouse tails for PCR amplification of the DNA fragment between a pair of primers, with the primer sequences listed in Table S3. The genotypes were then identified based on the bands of these amplification products after agarose gel electrophoresis.

### 5.14 Joint Retention and Biodistribution of SeNPs and M‐SeNPs

Mice that underwent DMM surgery were randomized to receive a single 20 µL intra‐articular injection of free DiD, DiD‐SeNPs, or DiD‐M‐SeNPs under anesthesia with pentobarbital sodium. Immediately after injection (t = day 0) and on days 1, 3, 7, 10, and 14, mice were positioned supine in the IVIS imaging system and fluorescence radiance (p s^−^^1^ cm^−^^2^ sr^−^^1^) was quantified from a fixed circular ROI over each knee after background subtraction using Living Image software (Version 4.7). At 24 h, mice were euthanized and ex vivo legs, livers, spleens, kidneys, hearts, and lungs were harvested and imaged to assess systemic distribution.

### 5.15 Behavioral and Functional Assessments

Baseline behavioral measurements were recorded prior to injection. All post‐injection tests were conducted by an experimenter blinded to the treatment groups. Pain‐related behaviors and motor function were evaluated by assessing mechanical allodynia, gait asymmetry, and motor coordination. Mechanical hypersensitivity was quantified on the plantar surface of the operated hind paw using calibrated von Frey filaments. Motor coordination and balance were tested using a rotarod apparatus (Sansbio, SA102) operated in an accelerating mode. The performance was quantified by recording the latency‐to‐fall. Three independent trials were conducted per mouse with a 40‐min recovery period between sessions. Gait analysis was performed using the CatWalk XT automated system (Noldus), where mice traversed a glass walkway voluntarily. Spatiotemporal parameters were recorded and compared between the ipsilateral and contralateral hind limbs.

### 5.16 Seahorse Metabolic Flux Analysis

A Seahorse XFe96 analyzer (Agilent) was used to measure ECAR (glycolysis surrogate) and OCR (mitochondrial respiration marker) according to Glycolysis Stress Test and MitoStress Test protocols. Serum‐starved FLS or RAW 264.7 macrophages were seeded into XF96 microplates and equilibrated in glucose‐free XF DMEM (pH 7.4) supplemented with 2 mM GlutaMAX in a CO_2_‐free incubator. ECAR was measured following sequential addition of glucose (10 mM), oligomycin(1.5 µM), and 2‐deoxy‐D‐glucose (50 mM). OCR was monitored at baseline and after injection of oligomycin (1.5 µM), carbonyl cyanide‐p‐trifluoromethoxyphenylhydrazone (FCCP, 1 µM), and rotenone/antimycin A (each at 0.5 µM). ECAR and OCR values were normalized to cell count per well (n = 6 biological replicates).

### 5.17 Statistical Analysis

All experiments were performed at least 3 times independently. Data are shown as the mean ± S.D. and analyzed via student's t‐test for two groups and one‐way ANOVA for multiple groups, followed by Tukey's test. GraphPad Prism 10.1.2 was used for all statistical analyses, with statistical significance was defined as *p* < 0.05.

## Author Contributions

**Yu‐Qing Zou**: methodology, data curation, writing – original draft, investigation, formal analysis. **Chen‐Hao Xu**: visualization, formal analysis. **Bo‐Wei Li**: validation, formal analysis. **Leung Chan**: methodology, visualization, data curation. **Yi‐Di Xu**: funding acquisition, methodology. **Han‐Bo Su**: software. **Rui Peng**: formal analysis. **Fang‐Ji Luo**: formal analysis. **Ya‐Chen Peng**: visualization. **Zhen‐Yan Li**: funding acquisition. **Vincent Kam Wai Wong**: funding acquisition. **Xiao‐Fei Zheng**: formal analysis. **Xing Ma**: funding acquisition. **Zhen‐Gang Zha**: funding acquisition, writing – review and editing, supervision. **Tianfeng Chen**: conceptualization, funding acquisition, resources, writing – review and editing. **Huan‐Tian Zhang**: writing – review and editing, visualization, conceptualization, project administration, resources.

## Funding

This work was financially supported by the Shenzhen Medical Research Fund (B2502029), Guangdong Basic and Applied Basic Research Foundation (2023B1515020007, 2025A1515012317 and 2024A1515010822), the National Science Fund for Distinguished Young Scholars (82225025), the National Natural Science Foundation of China (82502935, 82072470, and 22205079), Macau Foundation for Development of Science and Technology (0008/2024/RIA1), Science and Technology Projects in Guangzhou (2024A04J6408, 2024A03J0971 and 2025A03J4325), the Joint Funds of the National Natural Science Foundation of China (U24A6013), China Postdoctoral Science Foundation (2025M782023), K. C. Wong Education Foundation, and State Key Laboratory of Bioactive Molecules and Druggability Assessment, Jinan University (Grant No. SKLBMDA‐2024104).

## Conflicts of Interest

The authors declare no conflict of interest.

## Supporting information




**Supporting File**: advs78188‐sup‐0001‐SuppMat.docx.

## Data Availability

The data that support the findings of this study are available from the corresponding author upon reasonable request.
