# Supplementary material for: Metabolic‐Redox Remodeling of Inflammatory Synovium by Mannose‐Modified Selenium Nanoparticles for Osteoarthritis Therapy
Source: Adv Sci (Weinh). 2026 Oct 6:e78188. Online ahead of print. doi: 10.1002/advs.78188 (PMC13643119; doi:10.1002/advs.78188)
Supplement: Supplementary file 1 [file ADVS-9999-e78188-s001.docx]

#### **Supporting Information**

**Metabolic-Redox Remodeling of Inflammatory Synovium by Mannose-Modified Selenium Nanoparticles for Osteoarthritis Therapy**

Yu-Qing Zou, Chen-Hao Xu, Bo-Wei Li, Leung Chan, Yi-Di Xu, Han-Bo Su, Rui Peng, Fang-Ji Luo, Ya-Chen Peng, Zhen-Yan Li, Vincent Kam Wai Wong, Xiao-Fei Zheng, Xing Ma, Zhen-Gang Zha*, Tianfeng Chen*, Huan-Tian Zhang*

Yu-Qing Zou, Chen-Hao Xu, Bo-Wei Li, and Leung Chan contributed equally to this work.

Keywords: metabolic reprogramming, selenium nanoparticles, osteoarthritis, glycolysis, synovial inflammation, redox homeostasis

**Figure. S1. Single-cell RNA Sequencing Analysis Reveals Distinct Synovial Cell Subpopulations and Metabolic Alterations in OA.** (A) Schematic overview of the scRNA-seq workflow. (B) Uniform Manifold Approximation and Projection (UMAP) plots of the scRNA-seq dataset for non-OA (n=3) and OA (n=3) groups. (C) Proportional distribution of identified cell subpopulations within the synovial tissues of non-OA and OA groups. (D) Violin plots showing chronic inflammatory pathway scores calculated by AUCell in non-OA and OA synovium. (E) Heatmap showing relative expression (z-score) of glycolysis-related genes across synovial cell subpopulations identified by scRNA-seq.

**Figure. S2. Quantitative Analysis of GLUT1 Protein Expression Levels.** (A) Quantification of GLUT1 and TNF-α **expression levels based on** MFI, and the percentage of GLUT1^+^ cells within the TNF-α^+^ PDGFRα^+^ cell population (n=6). (B) Quantification of GLUT1 and TNF-α **expression levels based on** MFI, and the percentage of GLUT1^+^ cells within the TNF-α^+^ F4/80^+^ cell population (n=6). Quantitative data in (A, B) are shown as mean ± S.D., with statistical significance determined by Student's t-test. ***p* < 0.01, *****p* < 0.0001.

**Figure. S3. Elevated GLUT1 Protein Expression in Synovium of Fluorescent Reporter Mice with OA.** (A) Schematic illustration of lineage-tracing strategies and synovium collection from the DMM-induced OA mice model. FLS were labeled using tamoxifen-inducible *Prg4^GFPCreERt2^* mice, whereas macrophages were constitutively labeled in *Lyz2‑iCre;tdTomato* mice, enabling selective in vivo identification of GFP⁺ FLS and tdTomato⁺ macrophages. (B) Schematic diagram illustration of the strategy for generating *B6-Lyz2-iCre* mice*.* (C) Representative polymerase chain reaction (PCR) genotyping results confirming *B6-Lyz2-iCre* mice. Gel electrophoresis image displayed distinct DNA bands corresponding to the wild-type allele (lanes 1-3) and heterozygous allele (lanes 4-6). (D) Schematic diagram illustration of the strategy for generating *B6-G/R* mice*.* (E) Representative polymerase chain reaction (PCR) genotyping results confirming *B6-G/R* mice. Gel electrophoresis image displayed distinct DNA bands corresponding to the wild-type allele (lanes 1-3) and heterozygous allele (lanes 4-6). (F) Schematic diagram illustration of the strategy for generating *Prg4^GFPCreERt2^* mice*.* (G) Representative polymerase chain reaction (PCR) genotyping results confirming *Prg4^GFPCreERt2^* mice. Gel electrophoresis image displayed distinct DNA bands corresponding to the wild-type allele (lanes 1-3) and heterozygous allele (lanes 4-6). (H) Quantification of GLUT1 protein expression based on MFI and the percentage of GLUT1^+^ cells among GFP^+^ cells (n=6). (I) Quantification of GLUT1 protein expression based on MFI and the percentage of GLUT1^+^ cells among the tdTomato^+^ cells (n=6). Quantitative data in (H, I) are shown as mean ± S.D., with statistical significance determined by Student's t-test. ***p* < 0.01, *****p* < 0.0001.

**Figure. S4. Time-dependent effects of inflammatory stimuli on GLUT1 protein expression in FLS and RAW 264.7 Macrophages.** (A) GLUT1 protein expression levels in FLS. (B) Representative Western blot image showing GLUT1 protein expression in RAW 264.7 macrophages. (C) GLUT1 protein expression levels in RAW 264.7 macrophages. n=6. Quantitative data in (A, C) are shown as mean ± S.D., with statistical significance determined by one-way ANOVA. **p* < 0.05, ***p* < 0.01, *****p* < 0.0001.

**Figure. S5. The *SLC2A1*⁺ synovial cell subset exhibits enhanced glycolytic and inflammatory activity during synovial remodeling.** (A) UMAP plots visualizing four synoviocyte subpopulations (Syn_1 to Syn_4). (B) Proportional distribution of the four synoviocyte subpopulations in non-OA and OA groups. (C) Dot plot displaying the average expression level and percentage of cells expressing SLC2A1 across the four synoviocyte subpopulations. (D) Box plot showing the potency scores of each synoviocyte subpopulations. (E) Pseudotime trajectory analysis illustrating the inferred cell-state transitions among synoviocyte subpopulations. (F) Violin plot showing the expression level of *SLC2A1* in *SLC2A1*⁺ and *SLC2A1*⁻ subpopulations. (G-I) Violin plots showing glycolysis, Hallmark inflammatory response, IFNγ response and TNFα signaling pathway scores, calculated via AUCell, in *SLC2A1*^+^ and *SLC2A1*⁻ cell subpopulations. (J) Bar plot showing the enriched pathways of differentially expressed genes (DEGs) between *SLC2A1*^+^ and *SLC2A1*^-^ subsets. **p* < 0.05, ***p* < 0.01, *****p* < 0.0001.

**Figure. S6. Single-cell RNA Sequencing Reveals Impaired Selenoproteins in non-OA and OA Synovium.** (A) Feature plots illustrating oxidative stress pathway scores calculated via AUCell. (B) Violin plots showing the response to oxidative stress, ROS metabolic process, cellular response to ROS and Hallmark ROS pathway scores, calculated by AUCell, in non-OA and OA synovium. (C) Violin plots showing the expression levels of select selenoprotein-encoding genes, including *GPX2*, *DIO1*, *DIO2*, *SELENOH*, *SELENOO*, *TXNRD1*, *TXNRD2* and *TXNRD3*. (D) Heatmap of relative expression (z-score) of selenoprotein-related genes across *SLC2A1*^+^ and *SLC2A1*^-^ cell subsets identified by scRNA-seq. (E) Violin plots showing the response to oxidative stress, ROS metabolic process, cellular response to ROS and Hallmark ROS pathway scores, calculated via AUCell, in *SLC2A1*^+^ and *SLC2A1*^-^ cell subsets. (F) Violin plots showing the selenium metabolism and selenoproteins pathway scores, calculated via AUCell, in non-OA and OA synovium. (G) Violin plots showing the selenium metabolism and selenoproteins pathway scores, calculated by AUCell, in *SLC2A1*^+^ and *SLC2A1*^-^ cell subsets.

**Figure. S7. Quantitative Analysis of TXNRD1 and SELENOH Protein Expression Levels in vitro and in vivo.** (A) Quantification of TXNRD1 protein expression in IL-1β-induced FLS. (n=6). (B) Quantification of TXNRD1 and SELENOH protein expression assessed by MFI in *Prg4^GFPCreERt2^**.* (n=6). (C) Quantification of TXNRD1 and SELENOH assessed by MFI in *Lyz-iCre;tdTomato* (n=6). Quantitative data in (A, B, C) are shown as mean ± S.D., with statistical significance determined by one-way ANOVA (A) and Student's t-test (B, C). **p* < 0.05, ***p* < 0.01, ****p* < 0.001, *****p* < 0.0001.

**Figure. S8. Immunofluorescence Co-localization and Quantitative Analysis of GLUT1, TXNRD1, and SELENOH Protein Expression in Human Synovial Tissue and Mouse Knee Joints.** (A) Representative immunofluorescence co-staining images for GLUT1, TXNRD1, and SELENOH proteins in human synovial tissue. (B) Quantification of GLUT1, TXNRD1, and SELENOH protein expression assessed by mean fluorescence intensity (MFI) (n=6). (C) Representative immunofluorescence co-staining images for GLUT1, TXNRD1, and SELENOH proteins in mice knee joint tissue. (D) Quantification of GLUT1, TXNRD1, and SELENOH protein expression assessed by mean fluorescence intensity (MFI) (n=6). Quantitative data in (B, D) are shown as mean ± S.D., with statistical significance determined by Student's t-test. **p* < 0.05, ***p* < 0.01, ****p* < 0.001, *****p* < 0.0001.

**Figure. S9. Cellular Uptake and Endocytic Mechanisms of DiD-labeled SeNPs and M-SeNPs in RAW 264.7 Macrophages and FLS.** (A) Representative fluorescence microscopic images and flow cytometry histogram of cellular uptake of DiD-SeNPs and DiD-M-SeNPs at various concentrations (0.5, 2.5, 10, and 20 μM) in RAW 264.7 macrophages. (B) Representative fluorescence microscopic images and flow cytometry histogram of cellular uptake of DiD-SeNPs and DiD-M-SeNPs over a time gradient (2, 4, 6, and 8h) in RAW 264.7 macrophages. (C) Cell viability of cells treated with increasing concentrations of BAY-876 as assessed by CCK‑8 assay. (D, E) Glucose uptake capacity of FLS. (D, E) Glucose uptake capacity of FLS (D) and RAW 264.7 macrophages (E) detected by the 2-NBDG fluorescent probe after treatment with BAY-876. (F) Flow cytometry histogram of cellular uptake of DiD-M-SeNPs in FLS (left) and RAW 264.7 macrophages (right) pre-treated with GLUT1 inhibitor BAY-876. (G) Flow cytometry histogram of cellular uptake of DiD-M-SeNPs in FLS (left) and RAW 264.7 macrophages (right) pre-treated with free mannose. (H) Flow cytometry histogram of cellular uptake of DiD-M-SeNPs in RAW 264.7 macrophages pre-treated with specific inhibitors of endocytosis: chlorpromazine, wortmannin, and Filipin. Quantitative data in (C-E) are shown as mean ± S.D., with statistical significance determined by one-way ANOVA (C) and Student's t-test (D, E). ***p* < 0.01, ****p* < 0.001, *****p* < 0.0001.

**Figure. S10. Relative mRNA Expression Levels of *VCAM1*, *ICAM1*, *CXCL10*, *IL8*, *CCL2*, *CXCL2* and *CXCL3* in FLS Determined by qRT-PCR.** #1 Control, #2 IL-1β, #3 IL-1β + SeNPs, and #4 IL-1β + M-SeNPs. Quantitative data are shown as mean ± S.D., with statistical significance determined by one-way ANOVA. **p* < 0.05, ***p* < 0.01, ****p* < 0.001, *****p* < 0.0001, ns, not significant.

**Figure. S11. Analysis of M1 Macrophage Polarization Markers by qRT-PCR and Flow Cytometry.** (A) Relative mRNA expression levels of inflammatory cytokines (*Il1b, Il6, Tnfa*) and M1 macrophage markers (*Cd86, Nos2*) determined by qRT-PCR. (B) Flow cytometric analysis of CD86^+^ macrophage percentage within F4/80^+^ cells. #1 Control, #2 LPS, #3 LPS + SeNPs, and #4 LPS + M-SeNPs. Quantitative data in (A) are shown as mean ± S.D., with statistical significance determined by one-way ANOVA. **p* < 0.05, ***p* < 0.01, ****p* < 0.001, *****p* < 0.0001, ns, not significant.

**Figure. S12. Assessment of Intracellular ROS Levels and ΔΨm in RAW 264.7 Macrophages.** (A) Intracellular ROS levels in RAW 264.7 macrophages detected by DCFH-DA staining. (B) The mean DCF fluorescence intensity reflects the level of intracellular ROS (n=6). (C) ΔΨm changes in RAW 264.7 macrophages by JC-1 staining. (D) The Red/Green fluorescence intensity ratio (JC-1 aggregates/monomers) reflects the level of ΔΨm and is inversely proportional to mitochondrial depolarization (n=6). #1 Control, #2 LPS, #3 LPS + SeNPs, and #4 LPS + M-SeNPs. Quantitative data in (B, D) are shown as mean ± S.D., with statistical significance determined by one-way ANOVA. **p* < 0.05, ***p* < 0.01, ****p* < 0.001, *****p* < 0.0001, ns, not significant.

**Figure. S13. Proteomic Analysis Reveals Key Differentially Expressed Proteins and Enriched Pathways in IL-1β Induced FLS Treated with or without M-SeNPs.** (A) The protein expression levels of key inflammatory mediators (IL-6, CXCL1, CXCL3, CCL2, CCL20, VCAM-1, ICAM-1) and selenoproteins (TXNRD1, SELENOM, SELENOT, SELENOS, SELENOH) quantified from the proteomic data. Data are presented as log2 fold change. (B) Top enriched GO biological processes and KEGG pathways of differentially expressed proteins. The enrichment score -log10 P-value is plotted on the x-axis, and the corresponding enriched terms are listed on the y-axis. The size of the dot represents the count of differentially expressed proteins enriched in each term and pathway. (C) Protein expression abundance of PFKFB3 and TXNIP extracted from the proteomic data. Quantitative data in (C) are shown as mean ± S.D., with statistical significance determined by Student's t-test. ***p* < 0.01.

**Figure. S14. Glycolysis characterization in RAW 264.7 Macrophages under Different Treatments**. (A) Glucose uptake in RAW 264.7 macrophages measured using the fluorescent glucose analogue 2-NBDG. (B) Quantification of 2-NBDG MFI as an indicator of cellular glucose uptake. n=6. (C) Relative L-lactate levels in culture supernatants and intracellular lysates, normalized to the control group. n=3. (D) Real-time ECAR measurements during the glycolysis stress test, with sequential injections of glucose, oligomycin, and 2-DG. n=6. (E) Glycolysis ECAR (mpH/min/10⁵ cells), representing lactate production after glucose injection (non-glycolytic acidification subtracted). n=6. (F) Glycolytic capacity ECAR (mpH/min/10⁵ cells), representing maximal glycolytic activity after oligomycin treatment. n=6. (G) Real-time OCR measurements during the mitochondrial stress test, with sequential injections of oligomycin, FCCP, and rotenone/antimycin A (Rot/AA). n=6. (H) Basal respiration OCR (pmol/min/10⁵ cells), reflecting mitochondrial oxygen consumption under basal conditions. n=6. (I) Maximal respiratory capacity OCR (pmol/min/10⁵ cells), achieved after FCCP injection to uncouple oxidative phosphorylation. n=6. #1 Control, #2 LPS, #3 LPS + SeNPs, and #4 LPS + M-SeNPs. Quantitative data are shown as mean ± S.D., with statistical significance determined by one-way ANOVA. **p* < 0.05, ***p* < 0.01, *****p* < 0.0001, ns, not significant.

**Figure. S15. Quantitative Analysis of PFKFB3 Protein Expression Levels in Knee Joint Tissue of Transgenic Mice.** (A) Quantification of PFKFB3 protein expression assessed by MFI in *Prg4^GFPCreERt2^* mice joint tissues (n=6). (B) Quantification of PFKFB3 protein expression assessed by MFI in *Lyz2-iCre;tdTomato* mice joint tissues (n=6). Quantitative data are shown as mean ± S.D., with statistical significance determined by Student's t-test. ****p* < 0.001, *****p* < 0.0001.

**Figure. S16. Integrative Analysis of PFKFB3 Transcriptional Regulation and Correlation with ETS1 in FLS.** (A) Venn diagram identifying potential transcription factors (TFs) regulating PFKFB3. This Venn diagram illustrates the intersection of predicted PFKFB3 transcription factors derived from 4 sources: ChIP-Atlas, ENCODE, GTRD and hTFtarget databases, along with transcription factors identified by proteomic analysis. The overlapping region highlights common predicted or experimentally validated TFs, with ETS1 being specifically identified as a candidate regulator. (B) Box plots illustrating ETS1 expression levels in FLS and macrophages across NC and OA groups. (C) Protein expression abundance of ETS1 extracted from proteomic data. Quantitative data in (C) are shown as mean ± S.D., with statistical significance determined by Student's t-test. *****p* < 0.0001.

**Figure. S17. Other Fluorescence Images of OA Mice Knees over 14 Days after Intra-articular Injection of free DiD, DiD-SeNPs, and DiD-M-SeNPs.**

**Figure. S18. Representative H&E Staining of Major Organs in Different Treatment Groups following DMM Surgery.**

**Figure. S19. Heatmap Analysis of Complete Blood Count and Liver/Kidney Function Parameters for Evaluating in vivo Material Toxicity.** Each row represents a specific blood count or biochemical parameter (e.g., ALT, AST, BUN, CREA, WBC, RBC, PLT), and each column represents a different treatment group.

**Figure. S20. Immunofluorescence Co-localization and Quantitative Analysis of CD206 and F4/80 Protein Expression in Mice Knee Joints.** (A) Representative immunofluorescence co-staining of CD206 and F4/80 in knee joint synovium. Co-localized CD206⁺F4/80⁺ signals indicate anti-inflammatory M2 macrophages. (B) Quantification of the percentage of CD206^+^ F4/80^+^ macrophages in synovial tissue (n=6). C, cartilage; M, meniscus; S, synovium. Quantitative data in (B) are presented as mean ± S.D., with statistical significance determined by one-way ANOVA. ns, not significant.

**Table S1**. Informations of Antibodies Used for WB, IHC, and IF

| Antibody | Catalog Number | Company |
| --- | --- | --- |
| Anti-β-actin antibody | 3700 | CST, USA |
| [Anti-GLUT1 antibody](https://www.abcam.cn/products/primary-antibodies/glucose-transporter-glut1-antibody-epr3915-ab115730.html) | [ab115730](https://www.abcam.cn/products/primary-antibodies/glucose-transporter-glut1-antibody-epr3915-ab115730.html) | Abcam, USA |
| Anti-MMP13 antibody | ab39012 | Abcam, USA |
| Anti-PDGFR-α antibody | sc-398206 | Santa cruz, USA |
| Anti-TNF-α antibody | 17590-1-AP | Proteintech, USA |
| Anti-iNOS antibody | sc-7271 | Santa cruz, USA |
| Anti-F4/80 antibody | GB11027 | Servicebio, China |
| Anti-TRPA1 antibody | NB110-40763 | Novus, USA |
| Anti-TXNRD1 antibody | sc-28321 | Santa cruz, USA |
| Anti-SELENOH antibody | DF15312 | Affinity Biosciences, China |
| Anti-ICAM-1 antibody | [4915](https://www.cellsignal.cn/products/primary-antibodies/cd54-icam-1-antibody/4915) | CST, USA |
| Anti-VCAM-1 antibody | 13662 | CST, USA |
| Anti-CD206 antibody | ab64693 | Abcam, USA |
| Anti-TXNIP antibody | ab210826 | Abcam, USA |
| Anti-PFKFB3 antibody | A14764 | Abclonal, China |
| Anti-IL-1β antibody | BF8021 | Affinity Biosciences, China |
| Anti-ETS1 antibody | 14069 | CST, USA |

**Table S2**. Primers Used for the Detection of Indicated Genes by qRT-PCR

| Gene | Forward sequence | Reversed sequence |
| --- | --- | --- |
| *Mouse Nos2* | ACATCGACCCGTCCACAGTAT | CAGAGGGGTAGGCTTGTCTC |
| *Mouse Cd86* | TCTGCCGTGCCCATTTACAA | TGTGCCCAAATAGTGCTCGT |
| *Mouse Il1b* | TTCAGGCAGGCAGTATCACTC | GAAGGTCCACGGGAAAGACAC |
| *Mouse Il6* | GGCTAAGGACCAAGACCATCC | GCACTAGGTTTGCCGAGTAGA |
| *Mouse Tnfa* | CAGGCGGTGCCTATGTCTC | CGATCACCCCGAAGTTCAGTAG |
| *Mouse Actb* | ACCTTCTACAATGAGCTGCGT | TACATGGCTGGGGTGTTGAAG |
| *Human IL1B* | CTCGCCAGTGAAATGATGGCT | GTAGTGGTGGTCGGAGATTCG |
| *Human IL6* | GGTGTTGCCTGCTGCCTTCC | GTTCTGAAGAGGTGAGTGGCTGTC |
| *Human IL8* | CAGTTTTGCCAAGGAGTGCT | GTTTTCCTTGGGGTCCAGAC |
| *Human MMP13* | GCGGGAATCCTGAAGGAGAAT | TTTGCCAGTCACCTCTAAGCC |
| *Human ACTB* | CACAGAGCCTCGCCTTTGC | AATCCTTCTGACCCATGCCC |
| *Human CXCL1* | ACTCAAGAATGGGCGGAAAGC | TCAGGAACAGCCACCAGTGAG |
| *Human ICAM1* | CGACTGGACGAGAGGGATTG | GGAGAGCACATTCACGGTC |
| *Human VCAM1* | ATGTCAATGTTGCCCCCAGA | TGCTCCACAGGATTTTCGGA |
| *Human CXCL2* | CTTGTCTCAACCCCGCATCG | CCTCCTTCCTTCTGGTCAGTT |
| *Human CCL2* | GCAGAAGTGGGTTCAGGATTC | GGGTTGTGGAGTGAGTGTTCA |
| *Human CXCL10* | TGCTGCCTTATCTTTCTGACTCT | TGCTGATGCAGGTACAGCGT |

**Table S3**. Primers Used for Genetically Engineered Mice Genotyping

| Gene | Forward sequence | Reversed sequence |
| --- | --- | --- |
| *Prg4-CreERT*  *F1, R1* | TGACTATCTCAGGAATTCAAGCTG | CGCTTTTGACGCTGAAGTTAC |
| *Prg4-CreERT*  *F1, R2* | TGACTATCTCAGGAATTCAAGCTG | GAACTTCAGGGTCAGCTTGC |
| *Lyz2-iCre*  *F1, R1* | AGTGCTGAAGTCCATAGATCGG | CTGATTCTCCTCATCACCAGG |
| *Lyz2-iCre*  *F1, R2* | AGTGCTGAAGTCCATAGATCGG | GTCACTCACTGCTCCCCTGT |
| *B6-G/R*  *F1, R1* | TACGGCATGGACGAGCTGTAC | CCAACCTTTGTTCATGGCAG |
| *B6-G/R*  *F2, R2* | CAGCAAAACCTGGCTGTGGATC | ATGAGCCACCATGTGGGTGTC |
